# A functional motif of long noncoding RNA Nron against osteoporosis

**DOI:** 10.1038/s41467-021-23642-7

**Published:** 2021-06-03

**Authors:** Fujun Jin, Junhui Li, Yong-Biao Zhang, Xiangning Liu, Mingxiang Cai, Meijing Liu, Mengyao Li, Cui Ma, Rui Yue, Yexuan Zhu, Renfa Lai, Zuolin Wang, Xunming Ji, Huawei Wei, Jun Dong, Zhiduo Liu, Yifei Wang, Yao Sun, Xiaogang Wang

**Affiliations:** 1 Clinical Research Platform for Interdiscipline of Stomatology, The First Affiliated Hospital of Jinan University & Department of Stomatology, College of stomatology, Jinan University, Guangzhou, China; 2 Beijing Advanced Innovation Center for Big Data-Based Precision Medicine, School of Engineering Medicine, Beihang University, Beijing, China; 3 Department of Oral Implantology, School of Stomatology, Tongji University, Shanghai Engineering Research Center of Tooth Restoration and Regeneration, Shanghai, China; 4 Guangzhou Jinan Biomedicine Research and Development Center, National Engineering Research Center of Genetic Medicine, Institute of Biomedicine, College of Life Science and Technology, Jinan University, Guangzhou, China; 5 Shanghai Institute of Immunology, Department of Immunology and Microbiology, Shanghai Jiao Tong University School of Medicine, Shanghai, China; 6 Institute for Regenerative Medicine, Shanghai East Hospital, Frontier Science Center for Stem Cell Research, Shanghai Key Laboratory of Signaling and Disease Research, School of Life Sciences and Technology, Tongji University, Shanghai, China; 7 Department of Neurosurgery & China-America Institute of Neuroscience, Xuanwu Hospital, Capital Medical University, Beijing, China; 8 Zeki Biotechnology & Pharmaceutical Co. Ltd, Beijing, China

**Keywords:** Targeted bone remodelling, Osteoporosis

## Abstract

Long noncoding RNAs are widely implicated in diverse disease processes. Nonetheless, their regulatory roles in bone resorption are undefined. Here, we identify lncRNA Nron as a critical suppressor of bone resorption. We demonstrate that osteoclastic Nron knockout mice exhibit an osteopenia phenotype with elevated bone resorption activity. Conversely, osteoclastic Nron transgenic mice exhibit lower bone resorption and higher bone mass. Furthermore, the pharmacological overexpression of Nron inhibits bone resorption, while caused apparent side effects in mice. To minimize the side effects, we further identify a functional motif of Nron. The delivery of Nron functional motif to osteoclasts effectively reverses bone loss without obvious side effects. Mechanistically, the functional motif of Nron interacts with E3 ubiquitin ligase CUL4B to regulate ERα stability. These results indicate that Nron is a key bone resorption suppressor, and the lncRNA functional motif could potentially be utilized to treat diseases with less risk of side effects.

## Abstract (web summary)

LncRNAs are implicated in the pathogenesis of a number of diseases. Here, the authors show that the lncRNA Nron suppresses bone resorption, and show that delivery of a functional motif of Nron increases bone mass in mouse models of osteoporosis.

## Introduction

Osteoporotic fractures impart catastrophic effects on the health of elderly people^1–3^. Although current antiresorptive and anabolic osteoporosis drugs are effective to restraining osteoporotic fractures, both approaches have been associated with various side effects^4,5^. Thus, the identification of a novel drug target for the treatment and reversal of osteoporosis is warranted^6^. Long noncoding RNA (lncRNAs) have emerged as important regulators of gene expression or protein function by diverse molecular mechanisms at the transcriptional or post-transcriptional levels^7–9^. A growing number of lncRNAs have been implicated in various disease processes and are considered to have major diagnostic and therapeutic potential^10–13^. To identify functional lncRNAs, ascertaining the evolutionary conservation characters of lncRNAs was an efficient strategy^14–16^. To date, despite dozens evolutionary conserved lncRNAs has been identified, only a few of lncRNAs have been reported to contain conserved functional motifs^17–19^. In addition, the side effects of lncRNAs drugs in vivo could not be ignored. Whether the side effects of lncRNAs can be reduced by using functional motifs rather than full-length lncRNAs remains unclear. Thus, identifying the functional motifs that endow lncRNAs cellular activities is a strategy for the development of nucleic acid-based therapeutics.

Recently, the regulatory roles of lncRNAs in bone homeostasis have been recognized^20–23^. For instance, Bmncr^21^, Orlnc1^22^, and lnc-ob1^23^ are reported to involved in bone formation processes. Furthermore, our group reported that osteoblast-targeting delivery of lnc-ob1 ameliorates the bone loss phenotypes of OVX mice^23^. These reports highlight that lncRNAs may be potentially utilized as nucleic acid-based therapeutics for osteoporosis. Although few studies have reported that lncRNAs could regulate osteoclast differentiation in vitro^24–26^. Nevertheless, to the best of our knowledge, the biological roles of lncRNAs in controlling bone resorption in vivo and their therapeutic potential have yet to be determined^20,27^.

Here, we identify lncRNA Nron as a negative regulator of bone resorption. Osteoclastic-specific Nron knockout activates bone resorption. However, the overexpression of Nron in osteoclasts suppresses bone resorption. Pharmacological delivery of full-length Nron could effectively inhibit bone resorption in ovariectomized osteoporotic mice, although with obvious side effects. To further reduce the side effects of Nron during osteoporosis treatment, the functional motif of Nron was identified. Interestingly, the use of Nron functional motif significantly reduced the side effects while maintaining the same therapeutic effect as that of full-length Nron. Mechanistically, Nron interacts with E3 ligase CUL4B through the functional motif to regulate the stability of ERα in osteoclasts. Our study shows that Nron is a critical negative regulator of bone resorption and preliminarily reveals that lncRNA functional motifs could be utilized as alternative functional molecules in the treatment of diseases to reduce the possible pharmacological side effects of lncRNA-based drugs.

## Results

### Identification of Nron as a candidate regulatory lncRNA in osteoclasts

**Fig. 1:**
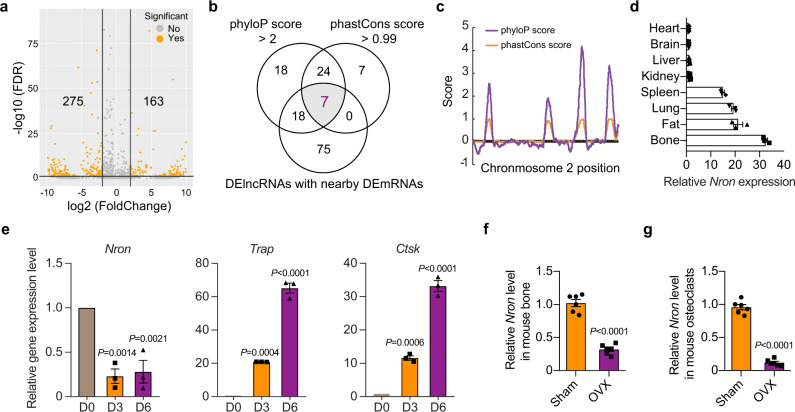
Identification of Nron as a candidate regulatory lncRNA in osteoclasts. **a** Volcano plots of the differential lncRNAs expression in undifferentiated BMMs and BMMs-derived osteoclasts. **b** Venn diagram representing the selection strategy for conserved lncRNAs. **c** PhastCons and PhyloP score of Nron. **d** Q-PCR analysis of Nron expression in eight types of C57BL/6 J mouse tissue, (*n* = 3/group). **e** Q-PCR analysis of *Nron*, *Trap*, and *Ctsk* gene expression levels during BMMs osteoclast differentiation, (*n* = 3 biologically independent experiments). **f** Q-PCR analysis of *Nron* expression in whole bone specimens (without bone marrow) from sham and ovariectomized (OVX) mice, (*n* = 6/group). **g** Q-PCR analysis of *Nron* expression in CTSK + osteoclasts isolated by microdissection from Sham and OVX mice, (*n* = 6/group). Significant differences between two groups were determined by unpaired Student’s *t*-test (two-tailed) and significant differences among multiple groups were determined by one-way ANOVA with Dunnett’s multiple comparisons test. Data are presented as the means ± s.e.m. Source data are provided as a Source Data file.

To identify the regulatory lncRNAs and lncRNA functional motifs involved in bone resorption, high-throughput RNA sequencing (RNA-seq) of mouse bone marrow-derived macrophages (BMMs) and BMMs-derived osteoclasts (OCs) was performed. A total of 438 lncRNAs with log2 (fold change) >1 were identified during mouse osteoclast differentiation, including 163 upregulated and 275 downregulated lncRNAs (Fig. 1a). Next, we performed PhastCons and PhyloP analysis^28^ to identify lncRNAs that contain evolutionarily conserved sequence motifs (Fig. 1b). We finally identified seven conserved lncRNAs (Fig. 1c, Supplementary Fig. 1a), of which Nron attracted our attention for the following reasons: (1) Nron is highly enriched in bone tissues (Fig. 1d, Supplementary Fig. 1b); (2) Nron contains four evolutionarily conserved motifs (Fig. 1c), of which two are located in the third exon (Supplementary Fig. 1a); (3) The conserved motifs of Nron do not overlap with known protein-coding genes and thus can be genetically knocked out (Supplementary Fig. 1a). Then, we verified the expression changes of *Nron* by quantitative real-time PCR (Q-PCR), the results showed that the expression levels of *Nron* were significantly reduced during BMMs osteoclast differentiation (Fig. 1e). Next, we evaluated the *Nron* expression in OVX mice, which is a typical experimental model that bone resorption increases^29^. The results showed that there was a significant decrease of *Nron* expression of ovariectomized mice when compared with sham-operated mice (Fig. 1f). In addition, we further explored the expression levels of *Nron* in osteoclasts isolated from mice bone specimens through laser-capture microdissection. We consistently detected lower levels of *Nron* expression in the osteoclasts isolated from OVX mice (Fig. 1g). Taken together, these findings suggest that Nron was a candidate regulator in osteoclasts.

### Activation of bone resorption predisposes Nron knockout mice to osteopenia

**Fig. 2:**
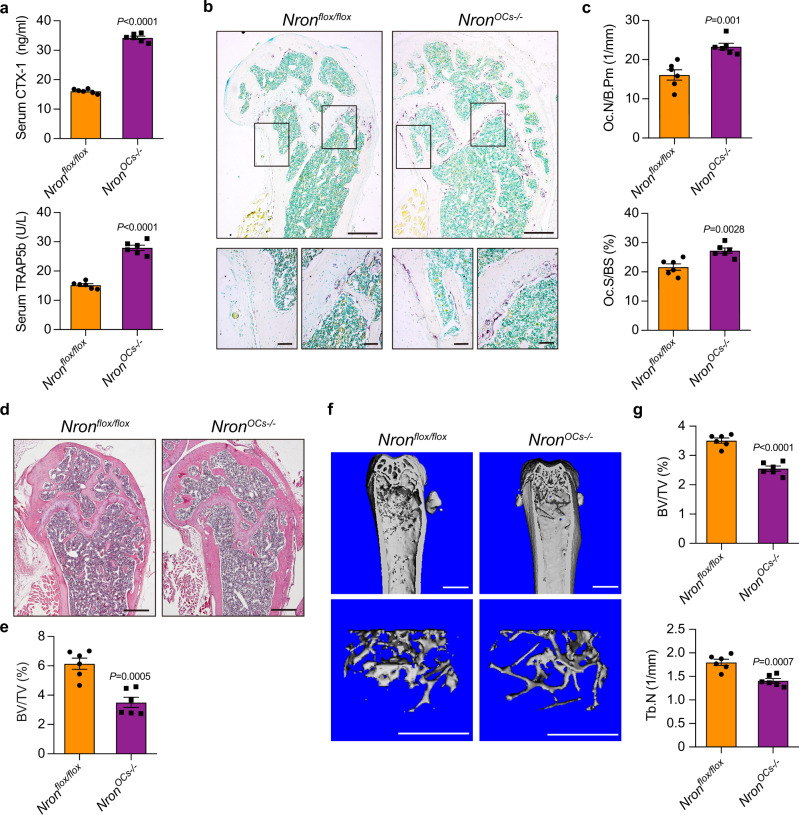
Osteoclast-specific Nron knockout activated bone resorption in mice. **a** Serum CTX-I and TRAP5b levels of 6-month-old female *Nron* ^*flox/flox*^ littermates and *Nron* ^*OCs−/−*^ mice analyzed by ELISA, (*n* = 6/group). **b** Representative TRAP staining images of femurs from 6-month-old female *Nron* ^*flox/flox*^ littermates and *Nron* ^*OCs−/−*^ mice, (similar results were obtained in all mice, *n* = 6/group). Scale bar: 500 μm (upper panels); 100 μm (lower panels). **c** Quantification of osteoclastic metrics in subepiphyseal region of femurs in **b**: Oc.N/B.Pm osteoclast number per bone perimeter, Oc.S/BS osteoclast surface per bone surface, (*n* = 6/group). **d** Representative H&E staining images of femurs from 6-month-old female *Nron* ^*flox/flox*^ littermates and *Nron* ^*OCs−/−*^ mice, (similar results were obtained in all mice, *n* = 6/group). Scale bar: 500 μm. **e** Histological measurements of BV/TV values in **d**, (*n* = 6/group). **f** Representative μCT images showing the 3D bone structures of the femurs from 6-month-old female *Nron* ^*flox/flox*^ littermates and *Nron* ^*OCs−/−*^mice, (similar results were obtained in all mice, *n* = 6/group). Scale bar: 1 mm (all panels). **g** μCT measurements of BV/TV and Tb.N in femurs from 6-month-old female *Nron* ^*flox/flox*^ littermates and *Nron* ^*OCs−/−*^ mice, (*n* = 6/group). Data are presented as the means ± s.e.m. Significant differences between two groups were determined by unpaired Student’s *t*-test (two-tailed). Source data are provided as a Source Data file.

Next, to explore whether Nron could modulate bone resorption in vivo, we generated osteoclast-specific Nron knockout mice (*Nron* ^*OCs−/−*^) by crossing *Nron* ^*flox/flox*^ mice with Ctsk-Cre mice (Supplementary Fig. 2a, b). The expression levels of *Nron* were significantly reduced in osteoclasts isolated by laser-capture microdissection from *Nron* ^*OCs−/−*^ mice, which indicated that Ctsk-Cre could successfully mediate knocking out of Nron in osteoclasts (Supplementary Fig. 2c, d). The upregulation of serum bone-resorption biomarkers^30^, CTX-I and TRAP5b, in *Nron* ^*OCs−/−*^ mice indicated that osteoclast-specific Nron knockout enhances bone-resorption activities of *Nron* ^*OCs−/−*^ mice (Fig. 2a). Meanwhile, the increase in the number of osteoclasts in the femurs of *Nron* ^*OCs−/−*^ mice was confirmed by tartrate-resistant acid phosphatase (TRAP) staining (Fig. 2b). In addition, the osteoclastic parameters Oc.N/B.Pm and Oc.S/BS was significantly increased in *Nron* ^*OCs−/−*^ mice (Fig. 2c). Furthermore, H&E staining showed that the bone volume of *Nron* ^*OCs−/−*^ mice was lower than *Nron* ^*flox/flox*^ littermates (Fig. 2d, e). Quantitative microtomography (μCT) analysis of the femurs showed that *Nron* ^*OCs−/−*^ mice displayed lower trabecular bone mass as compared with *Nron* ^*flox/flox*^ littermates (Fig. 2f). Meanwhile, the bone volume per tissue volume (BV/TV) and trabecular number (Tb.N) were significantly lower in the femurs of *Nron* ^*OCs−/−*^ mice (Fig. 2g). While there was no statistically significant change in cortical bone thickness in the femurs of 6-month-old *Nron* ^*OCs−/−*^ mice (Supplementary Fig. 2e, f). Furthermore, no changes in bone formation rates were observed in *Nron* ^*OCs−/−*^ mice base on dynamic histomorphometry measurements (Supplementary Fig. 2g, h). Thus, these results indicate that osteoclast-specific Nron knockout increases osteoclast numbers, and is associated with lower bone mass in vivo.

### Osteoclasts overexpressing Nron enhances bone mass due to suppressed bone resorption

**Fig. 3:**
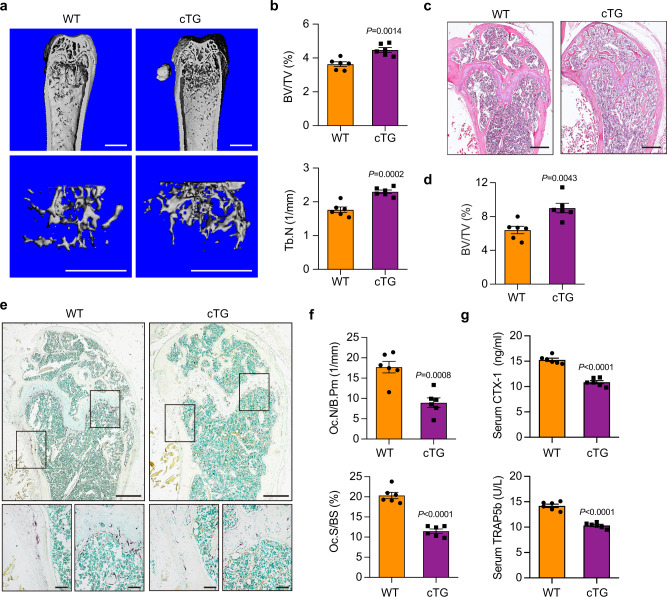
Increased bone mass in Nron cTG mice due to suppressed bone resorption. **a** Representative μCT images showing the 3D bone structures of femurs from 6-month-old female WT littermates and Nron cTG mice, (similar results were obtained in all mice, *n* = 6/group). Scale bar: 1 mm (all panels). **b** μCT measurements of BV/TV and Tb.N in femurs from 6-month-old female WT littermates and Nron cTG mice, (*n* = 6/group). **c** Representative H&E staining images of femurs from 6-month-old female WT littermates and Nron cTG mice, (similar results were obtained in all mice, *n* = 6/group). Scale bar: 500 μm. **d** Histological measurements of BV/TV values in **c**, (*n* = 6/group). **e** Representative TRAP staining images of femurs from 6-month-old female WT littermates and Nron cTG mice, (similar results were obtained in all mice, *n* = 6/group). Scale bar: 500 μm (upper panels); 100 μm (lower panels). **f** Quantification of osteoclastic metrics Oc.N/B.Pm and Oc.S/BS in subepiphyseal region of femurs in **e**, (*n* = 6/group). **g** Serum CTX-I and TRAP5b levels of 6-month-old female WT littermates and Nron cTG mice analyzed by ELISA, (*n* = 6/group). Data are presented as the means ± s.e.m. Significant differences between two groups were determined by unpaired Student’s *t*-test (two-tailed). Source data are provided as a Source Data file.

Next, to further determine whether osteoclast Nron overexpression inhibits bone resorption and enhances bone mass in vivo, we constructed an osteoclastic Nron transgenic mice (Nron cTG) derived from a Ctsk promoter (Supplementary Fig. 3a). The overexpression of *Nron* was verified by Q-PCR in osteoclasts isolated by laser-capture microdissection from wild-type (WT) and Nron cTG mice (Supplementary Fig. 3b, c). The results of μCT analysis showed that trabecular bone mass was significantly increased in the femurs of Nron cTG mice as compared to WT littermates (Fig. 3a). In addition, BV/TV and Tb.N significantly increased in Nron cTG mice (Fig. 3b). H&E staining also showed that the bone volume of the Nron cTG mice was remarkably enhanced (Fig. 3c, d). However, no statistical changes in cortical bone thickness were observed in the femurs of 6-month-old Nron cTG mice (Supplementary Fig. 3d, e). Furthermore, dynamic histomorphometry measurements did not detect any statistical changes in bone formation rates in the Nron cTG mice (Supplementary Fig. 3f, g). In addition, bone histomorphometric analysis showed that the number of TRAP+ osteoclasts significantly decreased in bone sections of the Nron cTG mice compared to their WT littermates (Fig. 3e). The osteoclastic parameters Oc.N/B.Pm and Oc.S/BS were decreased in the Nron cTG mice (Fig. 3f). The levels of serum bone-resorption biomarkers CTX-I and TRAP5b were also decreased in the Nron cTG mice (Fig. 3g). Taken together, these results showed that Nron is a negative regulator of bone resorption in vivo.

### Nron upregulates osteoclast ERα expression level

**Fig. 4:**
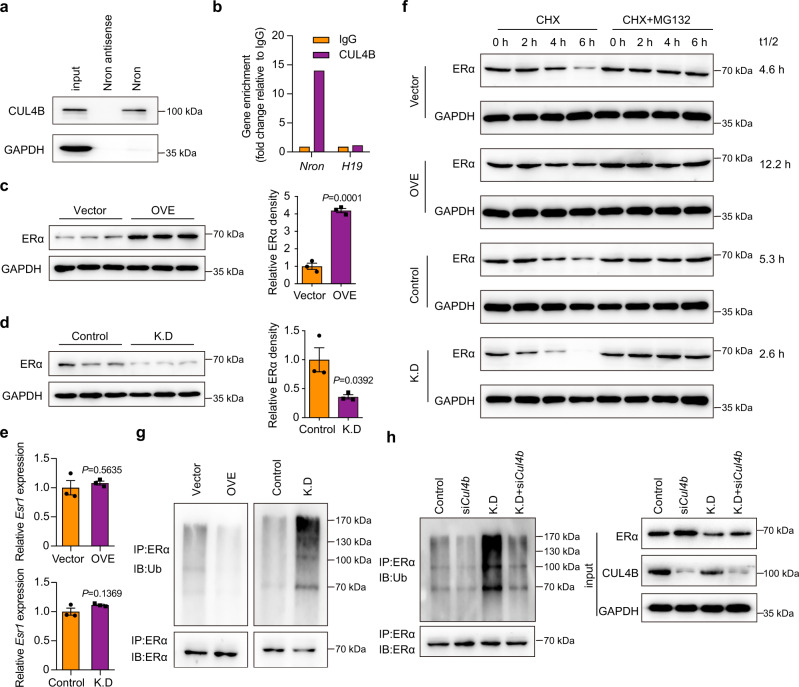
Nron regulates ERα stability in osteoclasts. **a** RNA pull-down assay and **b** RNA immunoprecipitation assay in mouse osteoclasts showed specific interaction between Nron and ubiquitin E3 ligase CUL4B. The experiment was repeated three times independently with similar results. **c, d** Expression of ERα in osteoclasts with *Nron* overexpression (OVE) or knockdown (K.D) analyzed by western blot (WB). The graph shows the relative ERα density (ERα/GAPDH levels, *n* = 3 biologically independent experiments). **e** Expression of *Esr1* gene in osteoclasts with *Nron* overexpression or knockdown analyzed by Q-PCR, (*n* = 3 biologically independent experiments). **f** The protein stability (t1/2) and the proteasome-mediated degradation of ERα assessed by measuring the decline of protein levels after CHX (10 μg/ml) or CHX (10 μg/ml) + MG132 (20 μM) treatment in osteoclasts. The experiment was repeated three times independently with similar results. **g** The ubiquitination of ERα in *Nron* overexpression or knockdown osteoclasts was assessed by immunoprecipitation (IP) with anti-ERα antibody followed by ubiquitin WB analysis. The experiment was repeated three times independently with similar results. **h** Forty-eight hours after scramble or *Cul4b* siRNA transfection in *Nron* knockdown or control osteoclasts, the cells were treated with 20 μM MG132 for 6 h, then the abundance of ubiquitinated ERα was assessed by IP with anti-ERα antibody followed by ubiquitin WB analysis. The experiment was repeated three times independently with similar results. Data are presented as the means ± s.e.m. Significant differences between two groups were determined by unpaired Student’s *t*-test (two-tailed). Source data are provided as a Source Data file.

Nron has been reported to interact with several proteasome system components, among which CUL4B is a well characterized E3 ubiquitin ligase of estrogen receptor α (ERα)^31,32^. Considering that ERα is one of the critical signaling pathways that regulates bone resorption^33,34^. Thus, a series of experiments were conducted to verify whether Nron is involved in the ERα degradation process by interacting with CUL4B. First, the interaction of Nron with CUL4B was verified by RNA pull-down (Fig. 4a) and RNA immunoprecipitation assays (Fig. 4b). We then conducted overexpression and knockdown experiments to evaluate the influence of Nron on ERα expression in osteoclasts. Figure 4c shows that *Nron* overexpression significantly upregulates the protein expression levels of ERα in osteoclasts. In contrast, *Nron* knockdown significantly decreased the accumulation of the ERα protein in osteoclasts (Fig. 4d). Furthermore, Q-PCR analysis revealed that Nron does not regulate *Esr1* gene expression (Fig. 4e). Therefore, we further investigated whether Nron influences the protein stability of ERα. Figure 4f shows that the ERα protein was gradually degraded in the presence of a translation inhibitor cycloheximide (CHX). *Nron* overexpression significantly enhanced the stability of the ERα protein, and the half-lives (t_1/2_) of ERα changed from 4.6 h in the vector group to 12.2 h in the *Nron* overexpression group (Fig. 4f). Meanwhile, *Nron* knockdown caused ERα to become highly labile, as indicated by a change in the t_1/2_ of ERα from 5.3 h in the control group to 2.6 h in the *Nron* knockdown group (Fig. 4f). Proteasome and lysosome are well characterized components of the degradation pathway that controls ERα protein stability^35^. The degradation of ERα was completely blocked by the presence of proteasome inhibitor MG132 (Fig. 4f), while lysosome inhibitor chloroquine treatment could not block the degradation of ERα (Supplementary Fig. 4). Meanwhile, immunoprecipitation of ERα followed by ubiquitin WB analysis revealed that the overexpression of Nron inhibited the pool of ubiquitinated ERα (Fig. 4g). However, the ubiquitination of ERα significantly increased after knocking down Nron and identified by high-molecular weight WB bands (Fig. 4g). Then, we knocked down *Cul4b* by siRNA in both control and *Nron* knockdown osteoclasts (Fig. 4h) to determine whether CUL4B is responsible for Nron-mediated ERα stability. The elevated ubiquitination of ERα caused by Nron knockdown was reversed by knocking down *Cul4b* (Fig. 4h). Thus, these results indicate that Nron interact with E3 ubiquitin ligase CUL4B to modulates the stability of ERα in osteoclasts. Next, to determine if Nron mainly acts via ERα, we silenced the gene expression of *Esr1* in osteoclasts with siRNA (Supplementary Fig. 5a). Then, we evaluated the anti-osteoclastic effects of Nron on osteoclasts with *Esr1* knockdown. The results showed that knockdown *Esr1* blocked the anti-osteoclastic effects of Nron, as indicated by the increased TRAP+ osteoclast numbers (Supplementary Fig. 5b, c) and the enhanced bone-resorption activities (Supplementary Fig. 5d, e). Furthermore, restoring the *Esr1* expression in *Esr1* knockdown osteoclasts rescued the anti-osteoclastic effects of Nron (Supplementary Fig. 5). Taken together, these results indicated that ERα was a mediator of the effects of Nron in osteoclasts.

### Osteoclastic delivery of Nron increases bone mass in OVX mice

**Fig. 5:**
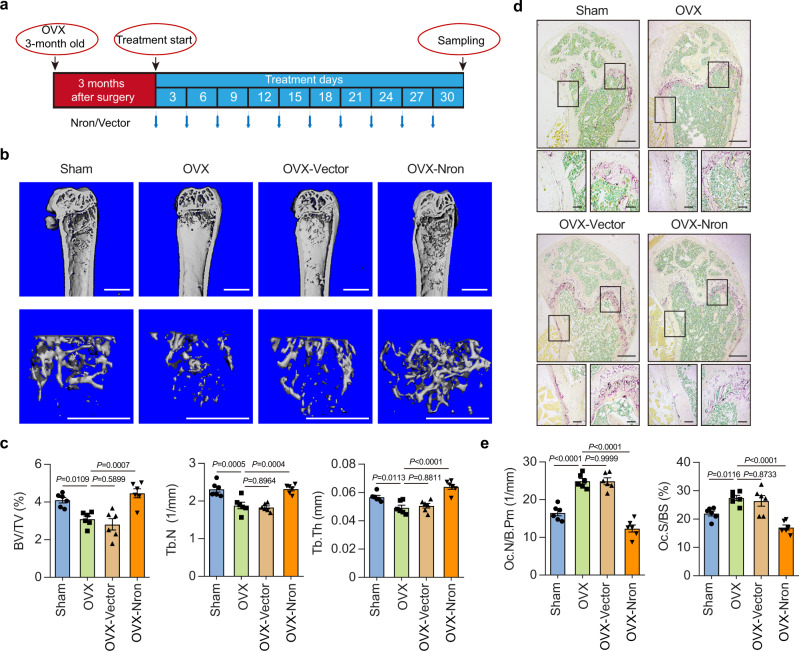
Osteoclastic delivery of Nron inhibits bone resorption of OVX mice. **a** Schematic diagram illustrating the experimental design of Nron treatment in OVX mice. **b** Representative μCT images showing the 3D bone structures of mice femurs from Sham, OVX, OVX-Vector, and OVX-Nron group, (similar results were obtained in all mice, *n* = 6/group). Scale bar: 1 mm (all panels). **c** μCT measurements of BV/TV, Tb.N, and Tb.Th in femurs from Sham, OVX, OVX-Vector, and OVX-Nron group, (*n* = 6/group). **d** Representative TRAP staining images of mice femurs from Sham, OVX, OVX-Vector, and OVX-Nron group, (similar results were obtained in all mice, *n* = 6/group). Scale bar: 500 μm (upper panels); 100 μm (lower panels). **e** Quantification of osteoclastic metrics Oc.N/B.Pm and Oc.S/BS in subepiphyseal region of femurs in **d**, (*n* = 6/group). Data are presented as the means ± s.e.m. The significant differences between Sham/OVX-Vector/OVX-Nron treatment group with OVX group were determined by one-way ANOVA with Dunnett’s multiple comparisons test. Source data are provided as a Source Data file.

Next, to determine the in vivo therapeutic potential of Nron in bone-resorption-related diseases, systemic administration of Nron by intravenous injection with the bone-resorption surface-targeting delivery system^36^ was performed in OVX mice (Fig. 5a). The Nron expression vectors were intravenously injected every three days. Thirty days after the first injection, the femurs were evaluated by μCT. Figure 5b shows that the bone mass of Nron-treated OVX mice significantly increased as compared to untreated OVX mice (OVX group). Meanwhile, the quantitative results of the BV/TV, Tb.N, and Tb.Th significantly increased in the femurs of Nron-treated mice (Fig. 5c). In addition, no significant variations in the bone mass were observed between the vector group with OVX group (Fig. 5b, c). Moreover, bone histomorphometric analysis with TRAP staining showed that the number of osteoclasts decreased in OVX mice after Nron treatment (Fig. 5d). Furthermore, osteoclastic parameters Oc.N/B.Pm and Oc.S/BS were significantly decreased with Nron treatment (Fig. 5e). Although Nron treatment did not affect mouse body weight (Supplementary Fig. 6a), it caused splenomegaly (Supplementary Fig. 6b, c). H&E staining showed the disappearance of the boundary between the white and red pulps and scattered megakaryocytes in hypertrophic spleens of Nron-treated mice (Supplementary Fig. 6d, e). Taken together, these in vivo therapeutic results further confirmed the inhibitory effect of Nron on bone resorption and preliminarily revealed its side effects.

### The functional motif of Nron interacts with CUL4B to regulate ERα ubiquitination

**Fig. 6:**
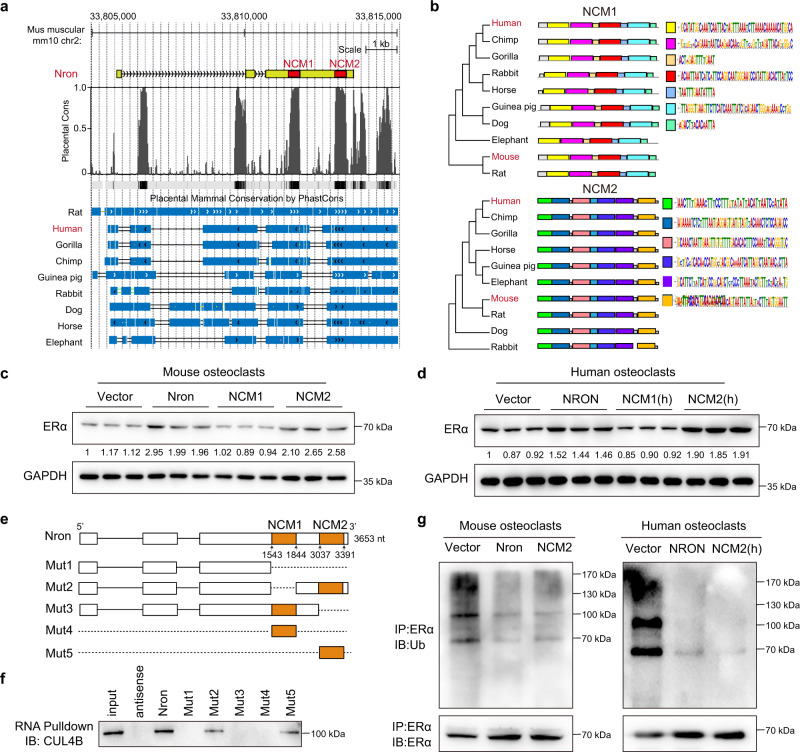
Functional motif of Nron interacts with Cul4b to regulate ERα ubiquitination. **a** Genome browser depiction of Nron and its conserved analogs in multiple species. **b** Evolutional tree of NCM1 and NCM2 in multiple species. **c** Expression of ERα in osteoclasts with Nron, NCM1, or NCM2 overexpression analyzed by WB. The experiment was repeated three times independently with similar results. **d** Expression of ERα in human THP-1 derived osteoclast with NRON, NCM1(h), NCM2(h) overexpression were analyzed by WB. The experiment was repeated three times independently with similar results. **e** Schematic diagram illustrating the mutation strategies of Nron. **f** Interactions of Nron and Nron mutants with CUL4B determined by RNA pull-down assay in mouse osteoclast lysates. The experiment was repeated three times independently with similar results. **g** The ubiquitination of ERα in Nron or NCM2 overexpression osteoclasts were assessed by immunoprecipitation (IP) with anti-ERα antibody followed by ubiquitin WB analysis. The experiment was repeated three times independently with similar results. Source data are provided as a Source Data file.

Toxicity is one of the decisive factors that limit the clinical transformation of drugs^37^. Therefore, we next dedicated our efforts to decrease the toxic effects caused by Nron. Considering that the Nron transcript is 3653-nt in length, we hypothesized that the observed splenomegaly in Nron-treated mice is caused by some uncharacterized motifs within Nron that could modulate unknown molecules and induce unexpected immune response activation. Recently reports have shown that lncRNAs contain conserved sequences that execute their functions^38,39^. Here, bioinformatics analysis revealed that there were two Nron conserved motifs (NCMs) in the third exon (Fig. 6a). Sequence similarity analysis indicated that both NCM1 and NCM2 are conserved in mammals (Fig. 6b), indicating that NCM1 and NCM2 were evolutional selected functional motifs of Nron. As we have indicated in Fig. 4 that Nron could modulate the stability of ERα in osteoclasts. Thus, we firstly cloned NCM1 and NCM2 and investigated their effect on the expression of ERα in osteoclasts. Figure 6c shows that the overexpression of NCM2 could significantly upregulates ERα expression levels in osteoclasts and is as efficient as that observed using full-length Nron. In addition, the immunofluorescence staining showed that osteoclasts with Nron or NCM2 overexpression contained more ERα proteins in the nuclei and cytoplasm (Supplementary Fig. 7a). Both WB (Fig. 6c) and immunofluorescence staining (Supplementary Fig. 7a) showed that NCM1 did not impart any significant regulatory effect on ERα at the protein level. Furthermore, to determine whether the human NRON (Supplementary Fig. 7b) and the two conserved human NRON motifs have the same effect as that of mouse Nron on the ERα, NRON, NCM1(h), and NCM2(h) were overexpressed in human osteoclasts. Consistently, NRON and NCM2(h) significantly increased the ERα levels in human osteoclasts (Fig. 6d). Thus, NCM2 works as a functional motif of Nron that could regulate ERα protein levels in osteoclasts. Next, we conducted mutational analysis of NCM1 and NCM2 to determine the interaction of Nron with CUL4B using an RNA pull-down assay (Fig. 6e). Interestingly, NCM2 alone was functional enough to interact with CUL4B, and the Nron mutant with NCM2 deletion abolished the interaction with CUL4B (Fig. 6f). Then, we further determined the influence of NCM2 on the ubiquitination of ERα in both mice and human osteoclasts. Figure 6g shows that NCM2 from both mice and human act as efficiently as the full-length of Nron in blocking ERα ubiquitination. Thus, these results indicated that NCM2 was a functional motif of Nron. Finally, we explored the effects of Nron and NCM2 on the ERα signaling in osteoclasts. TUNEL staining was performed to determine the effect of Nron or NCM2 on osteoclast survival. The percentage of TUNEL+ apoptotic osteoclasts is significantly increased in the Nron or NCM2 overexpressing osteoclasts with or without estradiol treatment (Supplementary Fig. 8a, b). Furthermore, the expression of the ERα downstream target FasL^33^ as well as apoptosis markers cleaved-Caspase3 and cleaved-PARP was significantly increased in the osteoclasts with Nron or NCM2 overexpression (Supplementary Fig. 8c, d). Collectively, these data reveal that Nron interacts with CUL4B via its functional motif NCM2 to control of ERα protein ubiquitination and modulate ERα signaling in osteoclasts.

### Osteoclast-targeting delivery of functional motif of Nron increases bone mass in osteoporotic mice

**Fig. 7:**
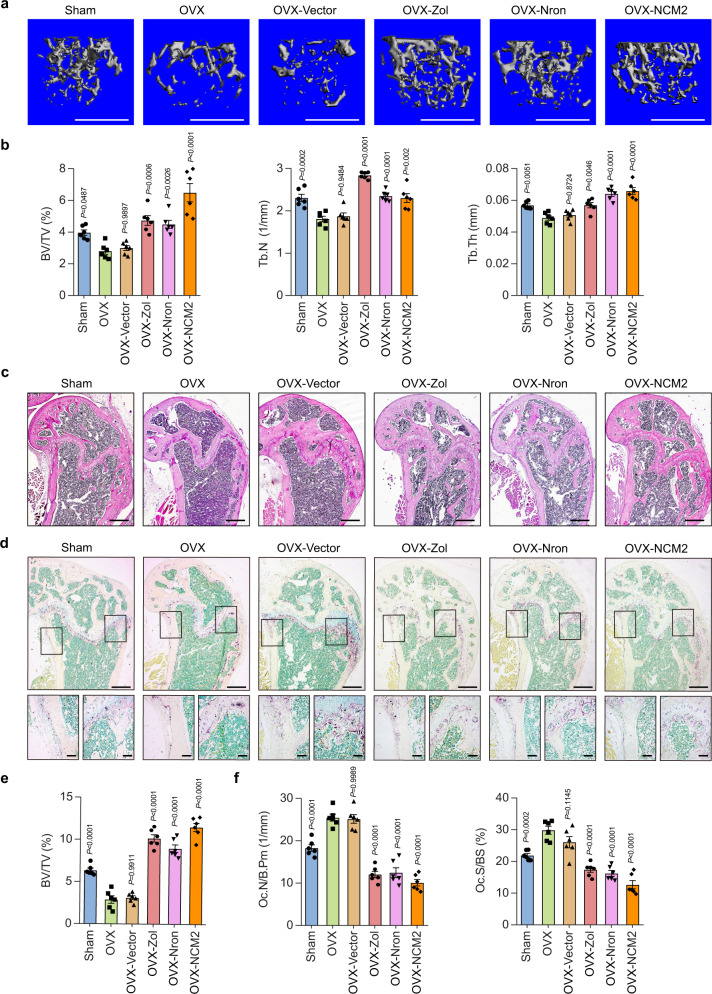
Osteoclast-targeting delivery of functional motif of Nron increases bone mass in osteoporotic mice. **a** Representative μCT images showing the 3D bone structures of femurs from Sham, OVX, OVX-Vector, OVX-Zol, OVX-Nron, and OVX-NCM2 group, (similar results were obtained in all mice, *n* = 6/group). Scale bar: 1 mm. **b** μCT measurements of BV/TV, Tb.N, and Tb.Th in femurs from Sham, OVX, OVX-Vector, OVX-Zol, OVX-Nron, and OVX-NCM2 group, (*n* = 6/group). **c** Representative H&E staining images of femurs from Sham, OVX, OVX-Vector, OVX-Zol, OVX-Nron, and OVX-NCM2 group, (similar results were obtained in all mice, *n* = 6/group). Scale bar: 500 μm. **d** Representative TRAP staining images of femurs from Sham, OVX, OVX-Vector, OVX-Zol, OVX-Nron, and OVX-NCM2 group, (similar results were obtained in all mice, *n* = 6/group). Scale bar: 500 μm (upper panels); 100 μm (lower panels). **e** Histological measurements of BV/TV values in **c**, (*n* = 6/group). **f** Quantification of osteoclastic metrics Oc.N/B.Pm and Oc.S/BS in subepiphyseal region of femurs in **d**, (*n* = 6/group). Data are presented as the means ± s.e.m. The significant differences between Sham/OVX-Vector/OVX-Zol/OVX-Nron/OVX-NCM2 treatment group with OVX group were determined by one-way ANOVA with Dunnett’s multiple comparisons test. Source data are provided as a Source Data file.

Then, how was the in vivo therapeutic potential and pharmaceutical safety difference between full-length Nron and its functional motif NCM2 is a critical issue needs to be determined. To this end, systemic administration of NCM2 with the osteoclast-targeting delivery system was performed on OVX mice 3 months after ovariectomy. Zoledronic acid (Zol), an antiresorption drug, was used as a positive control. The Nron/NCM2 expression vectors were intravenously injected every 3 days for 1 month. The results of μCT analysis showed that the trabecular bone mass of the femur (Fig. 7a) and fifth lumbar vertebrae (Supplementary Fig. 9a) of the NCM2-treated OVX mice significantly increased and showed similar efficiency as that using full-length Nron or Zol treatment. In addition, the quantitative results revealed that the BV/TV, Tb.N, and Tb.Th were significantly increased in the femur (Fig. 7b) and fifth lumbar vertebrae (Supplementary Fig. 9b) of the NCM2-treated mice. In contrast, the OVX mice treated with a blank vector (Vector group) or mice without treatment (OVX group) showed typical osteoporotic phenotypes with decreased BV/TV, Tb.N, and Tb.Th (Fig. 7a, b and Supplementary Fig. 9a, b). Meanwhile, H&E staining showed that the bone volume in the femur (Fig. 7c, e) and fifth lumbar vertebrae (Supplementary Fig. 9c, d) of NCM2-treated OVX mice was remarkably enhanced. Moreover, the bone histomorphometric analysis with TRAP staining showed that the bone resorption was strongly inhibited by NCM2 treatment in the femur (Fig. 7d) and fifth lumbar vertebrae bones (Supplementary Fig. 9e) of the OVX mice. Bone morphometric analysis also indicated that osteoclastic parameters Oc.N/B.Pm and Oc.S/BS were significantly decreased in OVX mice with NCM2 treatment (Fig. 7f and Supplementary Fig. 9f). Dynamic histomorphometry measurements showed that there were no statistically significant changes in bone formation rates with Nron or NCM2 treatment (Supplementary Fig. 9g, h). Thus, the therapeutic effects of Nron and NCM2 are mainly mediated by the inhibition of bone resorption. Most importantly, the spleen index and pathological H&E staining showed that the side effect of splenomegaly occurred in Nron treatment mice was significantly eliminated in the NCM2 treatment mice (Supplementary Fig. 10). Taken together, these in vivo therapeutic results further confirm that NCM2 is a functional motif of Nron and highlights the therapeutic potential of the Nron functional motif on bone-resorption-related bone loss.

### Functional motif of Nron improves cortical bone strength in osteoporotic mice

**Fig. 8:**
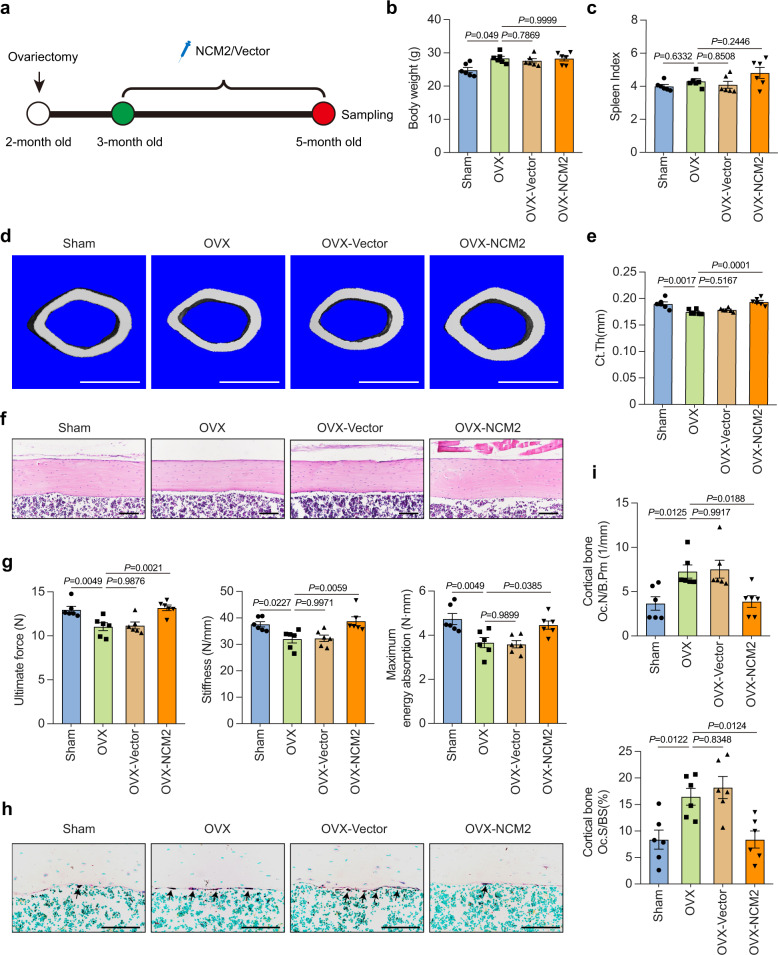
Functional motif of Nron improves cortical bone strength in OVX mice. **a** Schematic diagram illustrating the experimental design of NCM2 treatment in OVX mice. **b** Body weight of the mice from Sham, OVX, OVX-Vector, and OVX-NCM2 group, (*n* = 6/group). **c** Spleen index (spleen weight (mg) divided by body weight (g)) of mice from Sham, OVX, OVX-Vector, and OVX-NCM2 group, (*n* = 6/group). **d** Representative μCT images showing the 3D cortical bone structures of mice femurs from Sham, OVX, OVX-Vector, and OVX-NCM2 group, (similar results were obtained in all mice, *n* = 6/group). Scale bar: 1 mm. **e** μCT measurements of Ct.Th in femurs from Sham, OVX, OVX-Vector, and OVX-NCM2 group, (*n* = 6/group). **f** Representative cortical bone H&E staining images of femurs from Sham, OVX, OVX-Vector, and OVX-NCM2 group, (similar results were obtained in all mice, *n* = 6/group). Scale bar: 100 μm. **g** Biomechanical analysis of femur from mice as in **d**, (*n* = 6/group). The parameters measured include the ultimate force (N), stiffness (N/mm), maximum energy absorption (N·mm). **h** Representative TRAP staining images of the femur cortical from Sham, OVX, OVX-Vector, and OVX-NCM2 group, (similar results were obtained in all mice, *n* = 6/group). Scale bar: 100 μm. **i** Quantification of the endocortical surface osteoclastic metrics Oc.N/B.Pm and Oc.S/BS in (h), (*n* = 6/group). Data are presented as the means ± s.e.m. The significant differences between Sham/OVX-Vector/OVX-NCM2 treatment group with OVX group were determined by one-way ANOVA with Dunnett’s multiple comparisons test. Source data are provided as a Source Data file.

Cortical bone thickness and bone strength play a decisive role for resisting long bone fractures. Next, we explored if osteoclast-targeting delivery of NCM2 have potential protective effects on cortical bone thickness and bone strength in osteoporotic mice (Fig. 8a). The analysis of body weight (Fig. 8b) and spleen index (Fig. 8c) indicated that there was no significant side effects of 2 months NCM2 treatment in the OVX mice. The results of μCT analysis showed that two months NCM2 treatment significantly increased the cortical bone thickness of the OVX mice (Fig. 8d, e). Meanwhile, H&E staining consistently showed that the cortical bone thickness in the femur of NCM2-treated OVX mice was remarkably thicken (Fig. 8f). The cortical bone thickness changes were also reflected at biomechanical level. A three-point bending test in the femur highlighted the improvements in the bone strength of the OVX mice (Fig. 8g). Consistently, the bone histomorphometric analysis through TRAP staining showed that the endocortical bone resorption was strongly inhibited by NCM2 treatment of the OVX mice (Fig. 8h). Bone morphometric analysis of the endocortical surface also indicated that the osteoclastic parameters Oc.N/B.Pm and Oc.S/BS significantly decreased in OVX mice with NCM2 treatment (Fig. 8i). Taken together, these data show that 2 months NCM2 treatment exerts beneficial effects on the cortical bone thickness and the bone strength in osteoporotic mice and indicated the potential translational implication of lncRNA fragments, such as NCM2, to against bone resorptions in osteoporosis therapy.

## Discussion

Excessive bone resorption is one of the leading causes of osteoporosis. The antiresorptive drugs were currently widely used for the treatment of osteoporosis^40^. Although these antiresorptive drugs are effective in combating osteoporotic fractures, several side effects have been reported, such as bisphosphonate-related osteonecrosis of the jaw^41^. Therefore, it is necessary to identify novel targets for the treatment of osteoporosis^4^.

Over the past decade, the functions of lncRNAs have been considered as a fundamental aspect of biology^42^. Numerous studies have shown that lncRNAs are implicated in the pathophysiological processes of various diseases and thus may be utilized as therapeutic targets^43^. In vitro, the regulatory roles of lncRNA in osteoclastogenesis have been reported^20^. LINC00311 has been shown to activate Notch signaling and induce osteoclast differentiation^25^. LncRNA AK077216 upregulates the expression of NFATc1 to facilitate osteoclast differentiation^44^. LncRNA-Jak3 promotes MSU-induced osteoclast differentiation via the Jak3/Nfatc1/Ctsk signal axis^45^. However, the regulatory functions and therapeutic applications of lncRNAs for resisting bone resorption in vivo have yet to be determined. In this study, RNA-seq and lncRNA conservation analysis were performed to identify functional lncRNAs during osteoclast differentiation with conserved sequence motifs. Among seven lncRNAs that stood out in our analysis, based on gene structure and tissue expression levels, we focused on Nron for further functional analyses. Systematic evaluation using mouse models indicated that the conditional knockout of Nron in osteoclasts activated bone resorption, whereas the osteoclast-specific Nron transgenic mice exhibited increased bone mass with a significant decrease in bone-resorption activity. These results indicate that Nron is a negative regulator of bone resorption and may be potentially utilized as a therapeutic target for excessive bone resorption in osteoporosis. Thus, we further evaluated the in vivo therapeutic effects of Nron on osteoporosis. To achieve the targeted delivery of lncRNA molecules to bone tissues, we applied a recently developed bone-resorption surface-targeting nucleic acid delivery system^36^. The results of this study indicate that Nron targeting treatment significantly inhibits bone resorption and enhances bone mass of osteoporotic mice.

However, Nron therapy also showed apparent toxicity effects indicated by splenomegaly in Nron-treated mice. Immune response activation is one of the major issues that has hindered the translational application of nucleic acid-based therapy^46^. Considering that Nron is a 3653-nt long transcript with complicated secondary structures, we hypothesized that the splenomegaly phenotype of Nron-treated mice is caused by some uncharacterized motifs within Nron, which could induce unexpected immune response activation. Recent studies have suggested that specific conserved sequences in certain lncRNAs may serve as functional units^16,17^. Thus, to decrease the toxicity effects of lncRNAs, we dedicated our efforts to minimize the length of the lncRNA sequence by identifying the conserved functional motifs of Nron. Finally, we identified that the Nron 3’ end conserved motif 2 (NCM2) is the functional unit of Nron that inhibit bone resorption. The length of NCM2 is 354 nt, which is one-tenth of the full-length Nron. Interestingly, the bone-resorption inhibition activity of the NCM2 motif was as efficient as that of full-length Nron. Furthermore, splenomegaly or other obvious side effects were not observed in the NCM2-treated mice. Most importantly, 2 months NCM2 gene therapy significantly increased the cortical bone thickness and the bone strength of the OVX mice, thus indicating the potential translational implication of function motif of Nron in osteoporosis therapy.

Then, how Nron and its functional motif works is another critical issue. ERα is an essential modulator of osteoclast survival and bone resorption^33^. Cellular ERα levels are regulated by transcriptional and post-transcriptional networks^47^. Recently, lncRNA H19 and LINC01116 have been shown to regulate ERα expression in ERα+ breast cancer cells by functioning as competing endogenous RNA^48,49^. The present study has determined that Nron regulates ERα expression by modulating the proteasome degradation of the ERα in osteoclasts. In addition, we have further proven that Nron could directly interact with CUL4B through the functional motif NCM2 and control ERα ubiquitination. Thus, our results indicate that cellular ERα expression levels could be regulated by both lncRNAs or its motif at protein levels. The essential functions of ERα in the maintenance of bone mass under physiological conditions has been revealed by many articles^50,51^, but whether ERα has protective effects on bone loss induced by estrogen deficiency remains to be studied. In the present study, we found that enhancing the ERα expression in osteoclast could inhibit the bone resorption and against bone loss of the OVX mice (Supplementary Fig. 11). This result was consistent with the findings of Moon et al., they reported that Sirtuin 6 could protect against OVX-induced bone loss by enhancing the stability of the osteoclast precursor ERα proteins^47^. Thus, these findings suggest that increasing the expression of ERα in osteoclasts is a potential strategy to inhibit bone resorption and against bone loss in OVX mice. Meanwhile, the in vitro ERα loss of function studies indicated the anti-osteoclastic effects of Nron was mainly acts via ERα. Therefore, based on the regulation effects of Nron/NCM2 on ERα, and the phenotypes of the female Nron knockout and transgenic mice, as well as the antiresorptive effects of ERα, we believed that ERα was a major mechanism of Nron to regulate the bone resorptions in female mice.

In the present study, we mainly focused on the effects and therapeutic potential of Nron and its functional motifs on against bone resorption. Despite we observed that the application of Nron functional motifs eliminates the side effect of splenomegaly, the mechanisms by which Nron causes splenomegaly remain unclear and thus require further investigation. Meanwhile, it’s also valuable to know whether the essential sequence needed for normal function of the Nron functional motif be further narrowed.

In conclusion, we identify a lncRNA Nron that could inhibit bone resorption in vivo and evaluated the therapeutic potential of Nron and its functional motifs in the treatment of osteoporosis. Furthermore, we identify that the application of lncRNA gene therapy in vivo could cause non-negligible and unforeseeable side effects. Most importantly, our present study provides evidence that identifies the functional motif of lncRNA that may be utilized to eliminate the potential side effects of lncRNA-based gene therapy approaches. In summary, functional motifs of lncRNAs show great therapeutic potential to combat diseases, such as to inhibit of excessive bone resorption in osteoporosis.

## Methods

### Ethics statement

We have complied with all relevant ethical regulations for animal testing and research. All procedures for mouse studies were approved by the Ethics Committee of Tongji University School of Stomatology.

### Generation of osteoclast-specific Nron knockout mice

The Nron floxed mice on a C57BL/6 J genetic background were generated by Shanghai Research Center for Model Organisms (Shanghai, China) (Supplementary Fig. 2a). Briefly, the targeting vector contains 7.6 kb 5′-homologous arm, 4.3 kb flox region, PGK-Neo-polyA, 4.0 kb 3′-homolohous arm, and MC1-TK-polyA-negative screening marker was constructed and the linearized vector was electro-transfected into embryonic cells. After screening by nested long-range PCR, three positive clones were identified. Finally, after injected the positive clones into blastocysts of C57BL/6 J mice, four positive F0 chimeric mice were obtained. Then F0 chimeric mice were crossed with Rosa26-FlpE knock-in mice to remove the PGK-Neo cassette to generate the floxed F1 mice. Next, we crossed the Nron floxed mice with Ctsk-Cre mice (Supplementary Fig. 2b) (Cyagen Biosciences, Guangzhou, China) to obtain the osteoclast-specific Nron knockout mice. The genotyping PCR primers are Nron-flox (F: 5′-TGT CAC CAC CCA CCC TCG TA-3′, R: 5′-GAT GGC AAA CAG TGC CTC CA-3′, product size 269 bp), Ctsk-Cre (F: 5′-CAG CAT TGC TGT CAC TTG GTC-3′, R: 5′-ATT TGC CTG CAT TAC CGG TCG-3′, product size 569 bp). All animals used in this study were bred and maintained under specific pathogen-free (SPF) conditions with a temperature of 18–24 °C and humidity of 35–60% and 12 h light/dark cycle and free access to food and water.

### Generation of osteoclast-specific Nron transgenic mice

The osteoclast-specific Nron transgenic mice (Nron cTG) on a C57BL/6 J genetic background were generated by Cyagen Biosciences (Guangzhou, China) (Supplementary Fig. 3a). Briefly, pPB[Exp]-CAG > Ctsk-Nron vector was constructed by subcloning the mouse Ctsk promote and Nron cDNA into the pPB[Exp]-CAG plasmid. After verified the osteoclast-specific expression of the vectors in vitro, the linearized pPB[Exp]-CAG > Ctsk-Nron plasmid was then microinjected into C57BL/6 J oocytes. Next, the oocytes were transferred into pseudopregnant C57BL/6 J mice. Finally, two from 80 pups were identified as Nron cTG mice. The line >10 folds overexpression of Nron was used for further studies. The genotyping PCR primers are Ctsk-Cre (F: 5′-CAT TTA TGA CAA CTG CTC AGG GTC C-3′, R: 5′-TTC AGG GTC AGC TTG CCG TA-3′, product size 306 bp).

### Osteoclast-targeting therapy in OVX mice

Three-month-old female C57BL/6 J mice were performed with ovariectomy surgery (OVX) or received sham operation. Three months after surgery, these mice were randomly divided into six groups with six mice in each group, including Sham, OVX, OVX-Blank vector, OVX-Zoledronate (Zol, National Medical Term H20041967), OVX-Nron vector and OVX-NCM2 vector treatment group. The mice in Zol treatment group were intraperitoneal injection once with 80 μg/kg Zol. To achieve the targeted delivery of lncRNA molecules to bone tissues, we applied a recently developed Asp8-PU bone-resorption surface-targeting nucleic acid delivery system^36^, which contains two parts: Asp8 peptide, which has been reported as a bone-resorption surface-targeting peptide^52,53^, was used as the targeting part of the delivery system; polyurethane (PU) nanomicelles, which were able to encapsulate nucleic acids via electrostatic interactions^54^. For osteoclast-targeting therapy, 1 mg osteoclast-targeting delivery system Asp8-PU was dissolved in 1 ml saline through 10 min ultrasonic oscillation (SONICS) and then mixed with 1 mg vectors. The mice in OVX-Blank vector, OVX-Nron vector and OVX-NCM2 vector treatment groups was intravenous injected with 0.2 mg Asp8-PU loaded with vectors every 3 days for total 30 days. For sampling, the mice were anesthesia through intraperitoneal injection of 80 mg/kg pentobarbital sodium and performed with heart perfusion with 20 ml PBS solution and 20 ml 4% PFA. The right femur was collected for μCT and the left femur, heart, liver, spleen, lung, kidney, and brain were harvested for histopathology analysis. The Asp8-PU osteoclast-targeting delivery system was synthesized and characterized following the procedures described in our previously research^36^.

### Micro-CT analysis

Right femurs and the fifth lumbar vertebra collected form mice were scanned by micro-CT (μCT 50, Scanco Medical, Switzerland). The scanning resolution was set as 14.8 μm for one layer. The representative reconstruction image of femur and lumbar vertebra was taken, respectively, with the morphology of distal growth plate and foramevertebrale as reference. The bone mass analysis for trabecular bone in femur was limited to 100 slices upon distal femur growth plate. And bone mass analysis for trabecular bone in fifth lumbar vertebra was performed in 100 slices in vertebral body. For cortical bone analysis, the interested region was set as 50 slices (450–500 scanning layers upon distal femur growth plate) in midshaft of cortical bone. The threshold value of Micro-CT was set from 212 to 1000. The micro-CT image data were reconstructed and analyzed using Mimics software (v.13.0, Materialise NV).

### Three-point bending test

Femurs collected from mice of Sham, OVX, OVX-Vector, and OVX-NCM2 group were used for the three-point bending test through electronic universal testing machine (HENGYI, #HY-0230). Femurs with the physiological curvature facing up were fixed on a supporter with two fixed loading points with a 6 mm gap. A 1.0-mm-thick steel cross-bar plate, perpendicular to the long axis of the femurs and at the midpoint between two loading points, was used to apply a 1 N stabilizing preload. The bending load was applied at a constant displacement rate of 5 mm/min until fracture occurred. The ultimate force, stiffness and maximum energy absorption were determined from the load-deformation bending curve.

### H&E staining

The left femurs and the fifth lumbar vertebra of mice were decalcified in 10% EDTA solution for 28 days and the solution was renewed every 2 days. Femurs and other organs were dehydrated through automatic dehydrator (Microm STP120, Thermo). After embedded in paraffin, the tissues were sectioned at 4 μm thickness by rotary microtome (Microm HM325, Thermo). Then the sections were dewaxed in dimethylbenzene and graded ethanol, then immersed in water for 2 min. The sections were stained with Haematoxylin solution (BBI, #E607318-0206) for 1 min, differentiated in PBS for 5 min, then stained with Eosin solution (BBI, #E607318-0206) for 10 sec and rinsed with water. The sections were mounted with neutral resin after 10 min vitrification by dimethybenzene. Pictures were taken using the inverted microscope (DS-Ri1, Nikon).

### TRAP staining

After dewaxing, the paraffin sections were immersed in PBS for 2 min. The TRAP staining was performed using a TRAP Staining Kit (Sigma, #387 A) according to the manufacturer’s instructions. Briefly, the slices were stained with the staining solution for 15 min at 37 °C. Then slices were counterstained with 2% methyl green solution (Sigma, #M8020) for 10 min and rinsed with ethyl alcohol. The sections were mounted with neutral resin after 10 min vitrification by dimethybenzene. Pictures were taken using the inverted microscope (DS-Ri1, Nikon). Bone histomorphometric analyses for Oc.N/B.Pm and Oc.S/BS were performed on subepiphyseal region (Figs. 2, 3, 5, 7) or diaphyseal endocortical region (Fig. 8) in femur using Bioquant Osteo software (v.7.20.10, Bioquant Nashville) according to the standard procedures published by the American Society for Bone Mineral Research^55,56^.

### Double labeling

Double labelling analysis was performed according to our previous study^23^. Briefly, mice were respectively intraperitoneally injected with 10 mg/kg xylenol orange (Aladdin, #X101229-5G) and 20 mg/kg calcein (Sigma, #C0895-5G) 15 days and 1 days before sacrifice. After fixed in 4% PFA solution for 24 h, the femurs collected from mice was sectioned using a hard tissue sectioning and grinding system (EXAKT) at 20 μm thickness. The images were taken using the inverted microscope (Nikon, DS-Ri1). Bone dynamic histomorphometric analyses for MAR and BFR/BS were performed using Bioquant Osteo software (v.7.20.10, Bioquant Nashville) according to the standard procedures published by the American Society for Bone Mineral Research^55^.

### Laser-capture microdissection

Laser-capture microdissection was performed as previously described with some modifications^57^. In brief, the mice long bones were decalcified in RNase free 10% EDTA for 21 days and then embedded in optimal cutting temperature medium (SAKURA, #4583). Then, the series-frozen sections (10 μm) were prepared in a cryostat (Leica, # CM3050) at −24 °C. Adjacent sections were mounted on Frameslides (Leica, #11505190) and immunostained to identify the CTSK+ (Abcam, #ab19027,1:400) osteoclasts. Approximately 100–200 identified cells per specimens were collected and used for downstream RNA isolation using RNAprep Pure Micro Kit (TIANGEN, #DP420) according to the manufacturer’s instructions. The gene expression levels of *Nron* and osteoclast marker genes *Ctsk*, *Trap*, and *Mmp9* were quantified by Q-PCR assay. The gene expression levels were normalized with an internal housekeeping gene *Gapdh*. Gene-specific primer sequences are listed in Supplementary Table 1.

### High-throughput RNA-seq and data analysis

The BMMs were harvested before RANKL stimulation, and the osteoclasts were harvested after 6 days RANKL stimulation. The cell samples were collected in TRIzol Reagent (Invitrogen, #15596-018) and were sent to Gene Denovo Biotechnology Co. (Guangzhou, China) for RNA sequencing analysis using Illumina HiSeqTM 2500 (Illumina). Bowtie2 (v.2.2.6) was used for removing rRNA mapped reads. The remaining reads were then mapped to the reference genome of mm10 using Tophat2 (v. 2.1.1). The reconstruction of transcripts was carried out with Cufflinks (v.1.2.1) software and transcripts abundances were quantified by using RSEM (v1.3.1) software. A differential expression analysis was performed using EdgeR (v3.12.0). Genes that exhibited a standard *P* < 0.05 and log2 fold change >1 were filtered as differentially expressed genes.

### Target prediction and discovery of conserved lncRNAs

*Cis*-prediction was performed based on the differential expression transcripts. Differential expression mRNAs (DEmRNAs) were considered as *cis*-regulated target genes when the mRNA loci were within 10-kb of differential expression lncRNA (DElncRNAs) loci. The conservation of 200-bp sequences near each site of DElncRNAs was calculated, respectively, using the programs of PhastCons and PhyloP in software of PHAST (v.1.6.9), based on the known phylogenetic tree structure and phylo-HMM. The conserved lncRNAs with PhastCons score > 0.99 and PhyloP score > 2 were filtered.

### Highly conserved motifs identification

The highly conserved sequences in different species (Mouse, Rat, Human, Chimp, Gorilla, Rabbit, Horse, Guinea Pig, Dog, Elephant) was obtained from UCSC (http://genome.ucsc.edu) and Ensemble (http://asia.ensembl.org/Multi/Tools/Blast). The phylogenetic tree was constructed with neighbor-joining method using MEGA7 (v.7.0.21) software. The highly conserved motif was discovered with MEME (v.4.9.1) software (http://meme-suite.org/tools/meme).

### In vitro osteoclast differentiation

Mouse primary osteoclasts were differentiated from bone marrow-derived macrophages (BMMs) with the stimulation of M-CSF (Peprotech, #315-02) and RANKL (Peprotech, #315-11 C). Briefly, collect long bones from 8- to 12-weeks-old female C57BL/6 J mice, then flush out bone marrow using 1-ml syringe with serum-free α-MEM medium (Gibco, #12571-500). The bone marrow cells were suspended in growth medium (α-MEM + 10% FBS (Gibco, #10100-147)) and culture for one day. Then unattached cells were collected and adjust to 3 × 10^6^ cells/ml with α-MEM growth medium contain 50 ng/ml M-CSF and seeded in cell culture dish for one day. Subsequently, exchanged to differentiation medium (α-MEM + 10% FBS + 50 ng/ml M-CSF + 100 ng/ml RANKL) and renewal medium every two days for a total 6 days. Human osteoclasts were generated from human THP-1 cells (obtained from the Chinese Academy of Sciences Cell Bank, #TCHu57) with the stimulation of PMA (Sigma, #P1585), M-CSF and RANKL as pervious reported^58^. Briefly, THP-1 cells at a density of 5 × 10^5^ cells/ml were cultured in 1640 medium (Gibco, #11875-500) contain 10% FBS and stimulate with 100 ng/ml PMA for 2 days, then exchanged to differentiation medium (1640 + 10% FBS + 50 ng/ml M-CSF + 100 ng/ml RANKL) and renewal medium every two days for a total 14 days.

### Pit formation assay

The BMMs were plated on the Osteo Assay Surface Plate (Corning, #3988) and stimulated with osteoclast differentiation medium as described above. Pictures of resorption pits were taken using the inverted microscope (DS-Ri1, Nikon). The osteoclast resorption area per well was analyzed by image analysis freeware ImageJ (v.1.52a, National Institutes of Health, USA).

### Plasmid constructs

The full-length Nron was amplified by PCR using cDNA synthesized from BMMs as a template and cloned into the pcDNA3.1(+) vector (Invitrogen, #V79020). The Nron mutations were generated using the Q5 Site-Directed Mutagenesis Kit (NEB, #E0554S) and verified by sequencing. The pBI-CMV-Esr1 vector was constructed by IBSBio (Shanghai, China). The endotoxin-free plasmid was prepared using EndoFree Maxi Plasmid Kit (Tiangen, #DP120) according to the manufacturer’s instructions.

### Cell transfection

For gene overexpression or knockdown experiments, the cells were transfected with plasmids or siRNAs to mediated the gene overexpression or knockdown at day 4 after BMMs osteoclast differentiation and the osteoclasts were further cultured for 2 days in the M-CSF + RANKL osteoclast differentiation induction medium. Two days after transfections, the cells were collected for further analysis. The jet PRIME transfection reagent (Polyplus, #114-15) was used for plasmid or siRNA transfection. Briefly, 2 μg plasmid or 110 pmoles siRNA were added to 200 μl jet PRIME buffer and vortex 10 sec. Then, added 4 μl jet PRIME transfection reagent to the buffer, vortex 1 sec, spin down and incubate 10 min at room temperature (RT). Finally, added the transfection complex to the six-well plate and incubate for 6 h. Then change medium and culture for 48 h before sampling cells. The scramble (TTC TCC GAA CGT GTC ACG T), *Cul4b* siRNAs (AUA UAA GGU ACG AUG GAA GGA ACU G) and *Esr1* siRNAs (GGA GAA UGU UGA AGC ACA AUU) were synthesized by IBSBio (Shanghai, China).

### Protein stability assay

Cells were treatment with protein synthesis inhibitor Cycloheximide (MCE, #HY-12320, 10 μg/ml) alone or combined with proteasome inhibitor MG132 (MCE, #HY-13259, 20 μM) for 0, 2, 4, or 6 h. Cell lysates were collected and the protein levels of ERα were analyzed by WB. The ERα density was quantified using Quantity One Software (v4.62, Bio-Rad) and used to calculate the half-life time (t1/2) of the ERα proteins.

### Ubiquitination assay

Cells were treated with 20 μM MG132 for 6 h, and then rinsed twice with ice-cold PBS. The cells were lytic in Pierce IP Lysis Buffer (Pierce, #87787) containing protease inhibitors for 10 min on ice. Then, centrifugation at 12,000 × *g* for 10 min at 4 °C to collect the supernatant, and the protein concentrations was measured by BCA assays. Subsequently, 2 mg total proteins were precleared with normal mouse IgG (2 μg, Santa, #sc-2025) and protein A/G breads (Santa, #sc-2003) for 30 min at 4 °C. Then, incubated with ERα primary antibody (2 μg, Invitrogen, #MA1-12692) for 4 h at 4 °C, before being agitated with protein A/G agarose beads overnight at 4 °C. After washed complexes with ice-cold PBS five times, the immunoprecipitated proteins were collected in SDS loading buffer by heating at 100 °C for 5 min. The ubiquitination level of ERα was analyzed by WB with anti-Ubiquitin rabbit antibody.

### Immunofluorescence assay

Osteoclasts were fixed with 4% PFA for 10 min and then permeabilized with 0.1% Triton X-100 for 5 min. After blocked for 1 h in 5% BSA, the cells were incubated with the ERα primary antibody (Invitrogen, #MA1-12692, 1:100) at 4 °C overnight. Cells were rinsed three times with ice-cold PBS and incubated with Goat anti-Mouse Alexa Fluor 488 secondary antibody (Invitrogen, #A-11001, 1:1000) in 5% BSA for 1 h. Next, cells were rinsed three times with ice-cold PBS and incubated with 10 μg/ml DAPI (Invitrogen, #D1306) and Rhodamine Phalloidin (Invitrogen, #R415, 1:1000) for 30 min. Lastly, cells were rinsed three times with ice-cold PBS and visualized under a confocal laser scanning microscope (Zeiss, LSM700). To assay osteoclast apoptosis, we performed TUNEL staining. Briefly, the osteoclasts were transfected with Nron or NCM2 expression vectors at day 4 after osteoclast differentiation and the osteoclasts were further cultured for 2 days in M-CSF + RANKL differentiation medium in the presence or absence of 10 nM estradiol, then collected for TUNEL analysis. TUNEL assay was performed with a commercially available One Step TUNEL Apoptosis Assay Kit (Beyotime, #C1086) according to manufacturer’s instructions.

### Quantitative real-time PCR assay (Q-PCR)

The TRIzol Reagent (Invitrogen, # 15596-018) was used for total RNA isolation from cultured cells and RNAprep Pure Tissue Kit (Tiangen, #DP431) was used for total RNA isolation from liquid nitrogen homogenized bone tissues. The PrimeScript RT Reagent Kit with gDNA Eraser Kit (Takara, #RR047A) was used for cDNA synthesis. Q-PCR assays were conducted in a CFX96 Touch Real-Time PCR Detection System (Bio-Rad) using a TB Green Premix Ex Taq II Kit (Takara, #RR820A) according to the manufacturer’s instructions. Data was analyzed by CFX Manager 3.0 software (Bio-Rad). The gene expression levels were normalized with an internal housekeeping gene *GAPDH*. Gene-specific primer sequences are listed in Supplementary Table 1.

### Western blot assay

Cells were rinsed once with ice-cold PBS then scraped from plates using SDS lysis buffer (Beyotime, #P0013G) containing protease inhibitors (Beyotime, #P1005), and lytic at 100 °C for 10 min. Then, centrifugation at 12,000 × *g* for 10 min at 4 °C to collect the supernatant, and measurement the protein concentrations by BCA Protein Assay Kit (Beyotime, #P0012S). Proteins were separated by SDS-PAGE and transferred to PVDF membranes (Millipore, IPVH0010), after blocking with 5% w/v nonfat milk at RT for 1 h, the membranes were incubated with specific primary antibodies overnight at 4 °C. Subsequently, the membranes were incubated with HRP-conjugated secondary antibodies at RT for 1 h. Blots were visualized by chemiluminescence reagent (Millipore, #WBLUR0500). The band density was quantified using Quantity One Software (v4.62, Bio-Rad). The primary antibodies included anti-ERα (Invitrogen, #MA1-12692, 1:200), anti-CUL4B (Proteintech, #12916-1-AP, 1:1000), anti-Ubiquitin (CST, #43124, 1:1000), anti-FasL (abcam, #ab15285, 1:1000), anti-PARP (CST, #9532, 1:1000), anti-GAPDH (CST, #5174, 1:1000), anti-Caspase3 (CST, #9662, 1:1000), Anti-mouse IgG, HRP-linked Antibody (CST, #7076, 1:5000) and Anti-rabbit IgG, HRP-linked Antibody (CST, #7074, 1:6000). The complete unedited blots are shown in Source data.

### RNA pull-down assay

The RNA pull-down assay was performed using Pierce Magnetic RNA-Protein Pull-Down Kit (Pierce, #20164) according to the manufacturer’s instructions. Briefly, the Transcript Aid T7 High Yield Transcription Kit (Thermo, #K0441) was used for in vitro transcription of Nron or Nron antisense RNA from linearized plasmid, and the Pierce^TM^ RNA 3’ End Desthiobiotinylation Kit (Pierce, #20163) was used to attach biotinylated nucleotide to the RNA strand. Then, 100 pmol biotinylated RNA were incubate with 50 μl streptavidin magnetic beads 30 min at RT with rotation. Subsequently, 200 μg osteoclast lysate were added to the RNA-bound beads and incubate 2 h at 4 °C with rotation. Next, washing the protein-RNA-beads complex with wash buffer five times, then add 50 μl elution buffer to the complex and incubate 30 min at 37 °C with rotation. Finally, add SDS loading buffer to samples and heating for 5 min at 100 °C, which were then detected by WB.

### RNA immunoprecipitation assay

The RNA immunoprecipitation assay was performed using Magna RIP RNA-Binding Protein Immunoprecipitation Kit (Millipore, #17-701) according to the manufacturer’s instructions. Briefly, 115 μl RIP Lysis Buffer contain RNase inhibitor and protease inhibitor was added to each 15 cm plate cultured with osteoclast cells, after incubate on ice for 5 min the cell lysate was collect and frozen once at −80 °C. Then, mix 50 μl of magnetic beads with CUL4B antibodies (5 μg, Proteintech, #12916-1-AP) or negative control IgG (5 μg, Millipore, #PP64B) and incubate 30 min at RT with rotation. Subsequently, thaw the lysate quickly and centrifuge at 14,000 g for 10 min at 4 °C, and transfer 100 μl supernatant to the antibody-bound beads, incubate overnight at 4 °C with rotation. Next, washing the beads with RIP Wash Buffer five times, and digest the samples with proteinase K at 55 °C for 30 min. Finally, purify the RNA with phenol:chloroform:isoamyl alcohol and the enrichment of specific RNA were analyzed by Q-PCR assay.

### Statistical analysis

All data are presented as the mean ± s.e.m. Significant differences between two groups were determined by unpaired Student’s *t*-test (two-tailed). Significant differences between multiple groups were determined by one-way with Dunnett’s multiple comparisons test or two-way ANOVA with Sidak’s multiple comparisons test. *P*-value of less than 0.05 was considered statistically significant. GraphPad Prism version 8.0 was used for all statistical analyses.

### Reporting summary

Further information on research design is available in the Nature Research Reporting Summary linked to this article.

## Supplementary information

Supplementary Information

Reporting Summary

## Source data

Source Data

## Data Availability

The authors declare that the data supporting the findings of this study are available within the paper and its Supplementary information files. Any remaining data that support the results of the study will be available from the corresponding author upon reasonable request. The high-throughput RNA-seq data have been have been deposited in the Gene Expression Omnibus (GEO) under accession code GSE134457. A reporting summary for this article is available as Supplementary Information file. Source data are provided with this paper.
