# Supplementary material for: A functional motif of long noncoding RNA Nron against osteoporosis
Source: Nat Commun. 2021 Jun 3;12:3319. doi: 10.1038/s41467-021-23642-7 (PMC8175706; doi:10.1038/s41467-021-23642-7)
Supplement: Supplementary file 1 — Supplementary Information [file 41467_2021_23642_MOESM1_ESM.pdf]

Supplementary Information

A functional motif of long noncoding RNA Nron against osteoporosis

Jin et al.

Supplementary Information contains

Supplementary Figures 1–11

Supplementary Table 1

Sequences of Nron functional motif

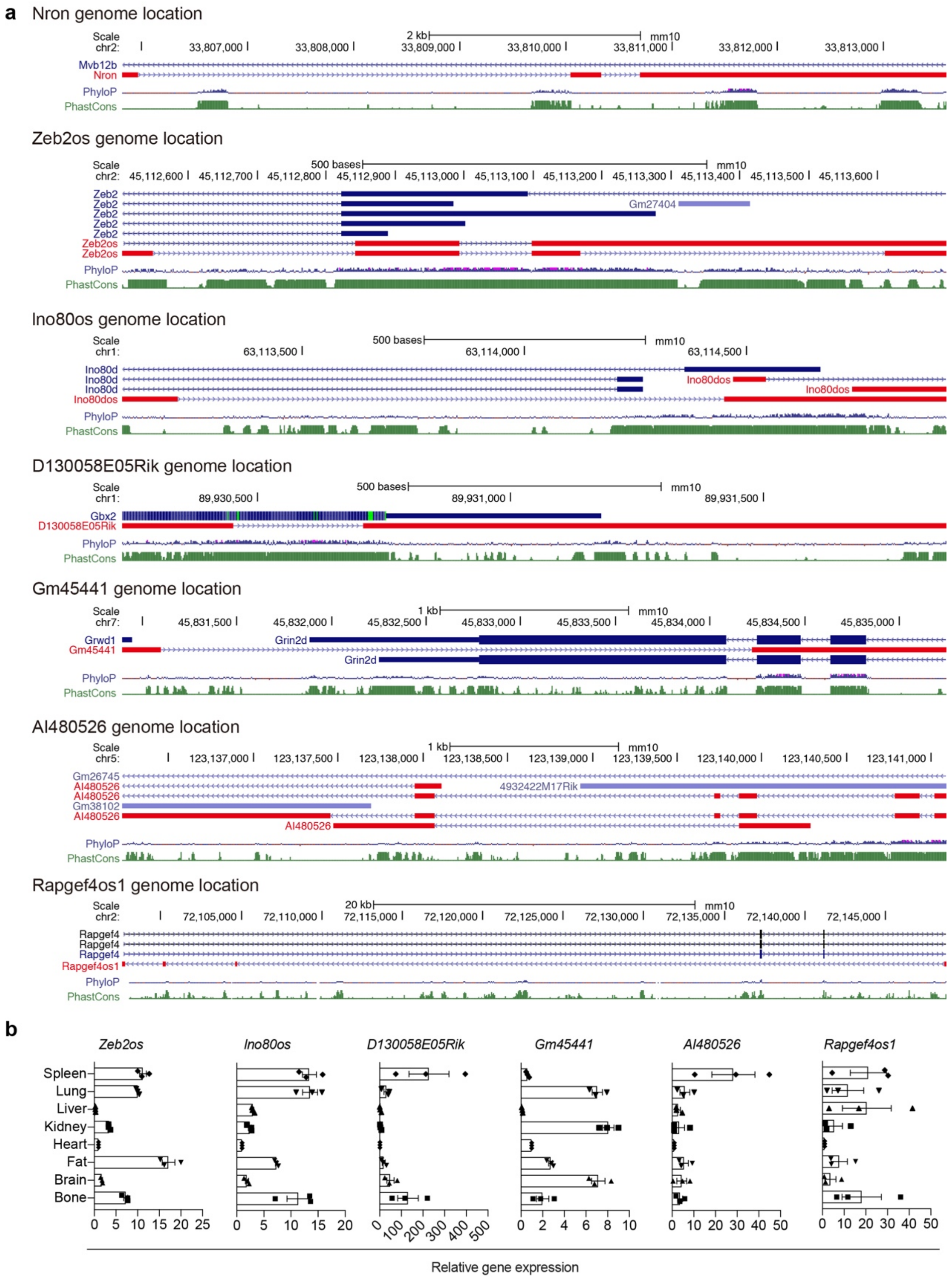

Supplementary Fig. 1 Genome location and tissue expression of selected conserved lncRNAs. (**a**) Gene organization and relative location of selected conserved lncRNAs in UCSC. (**b**) Q-PCR analysis of six selected conserved lncRNAs in eight types of C57BL/6J mouse tissue, (n=3/group). Data are presented as the means ± s.e.m. Source data are provided as a Source Data file.

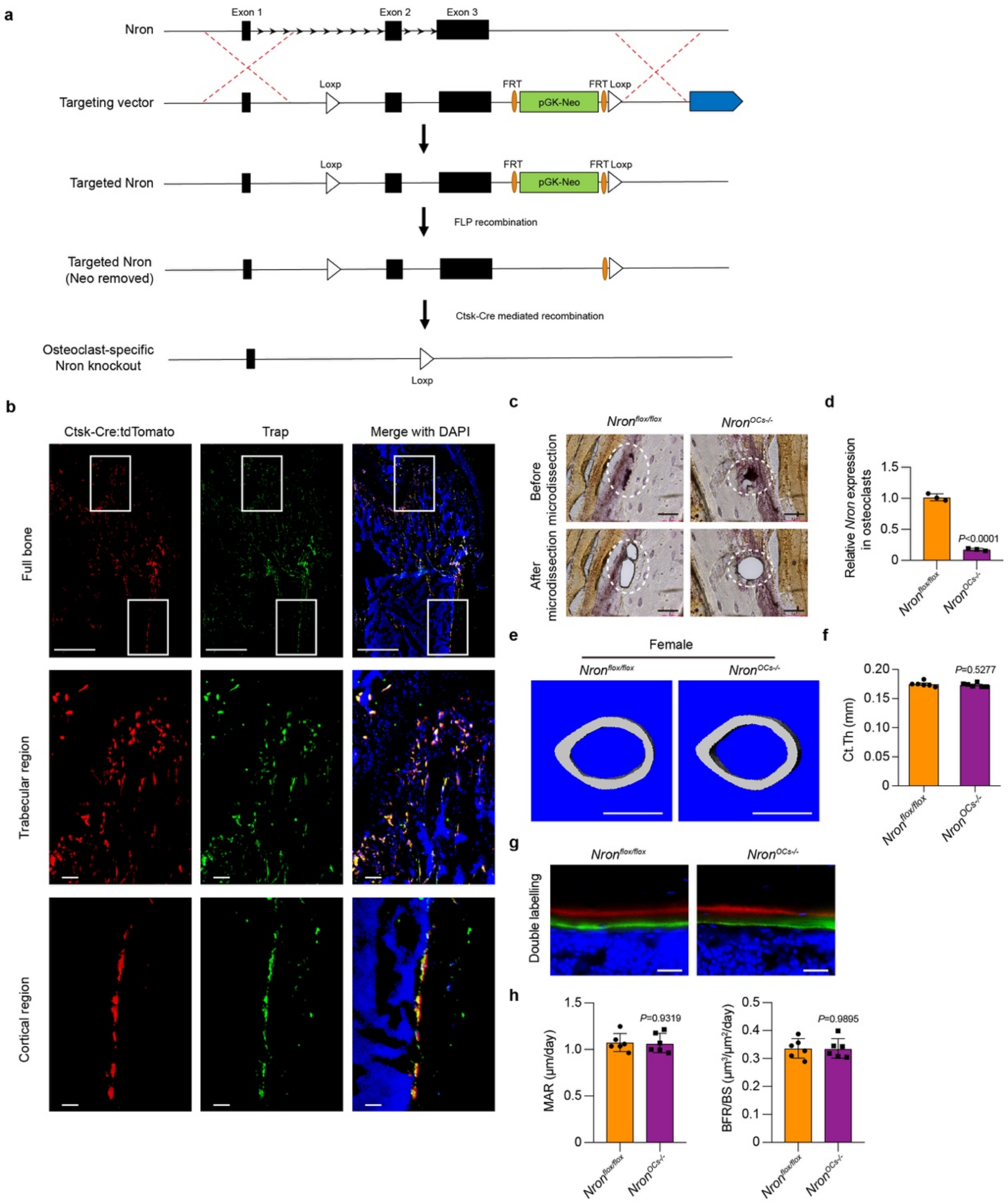

Supplementary Fig.2 See next page for caption

Supplementary Fig. 2 Generation and characterization of osteoclast-specific Nron knockout mice. (**a**) Schematic illustration of the generation of osteoclast-specific Nron knockout mice. (**b**) The femur bone sections from 7-weeks-old female Ctsk:tdTomato reporter mice were under immunofluorescence staining with Trap antibodies (Abcam, #ab191406, 1:100) to evaluate the osteoclast targeting specificity of the Ctsk-Cre, (similar results were obtained in all mice, n=6/group). Scale bar: 500 μm (upper panels); 200 μm (middle and lower panels). (**c**) Representative images of the TRAP+ osteoclasts in femur from 6-month-old female $Nron^{flox/flox}$ and $Nron^{OCs-/-}$ mice before and after the laser-capture microdissection, (similar results were obtained in all mice, n=3/group). Scale bar: 25 μm. (**d**) Q-PCR analysis of *Nron* expression in TRAP+ osteoclasts isolated by laser-capture microdissection from 6-month-old female $Nron^{flox/flox}$ and $Nron^{OCs-/-}$ mice, (n=3/group). (**e**) Representative μCT images showing the 3D cortical bone structures of the femurs from 6-month-old female $Nron^{flox/flox}$ and $Nron^{OCs-/-}$ mice, (similar results were obtained in all mice, n=6/group). Scale bar: 1 mm. (**f**) μCT measurements of Ct.Th in femurs from 6-month-old female $Nron^{flox/flox}$ and $Nron^{OCs-/-}$ mice, (n=6/group). **(g)** Representative images of bone formation rates of 6-month-old female $Nron^{flox/flox}$ and $Nron^{OCs-/-}$ mice as determined by double labeling of xylenol orange and calcein, (similar results were obtained in all mice, n=6/group). Scale bar: 20 μm. **(h)** Bone histomorphometric analysis of MAR and BFR/BS in femurs from 6-month-old female $Nron^{flox/flox}$ and $Nron^{OCs-/-}$ mice, (n=6/group). Data are presented as the means ± s.e.m. Significant differences between two groups were determined by unpaired two-tailed Student's t-test. Source data are provided as a Source Data file.

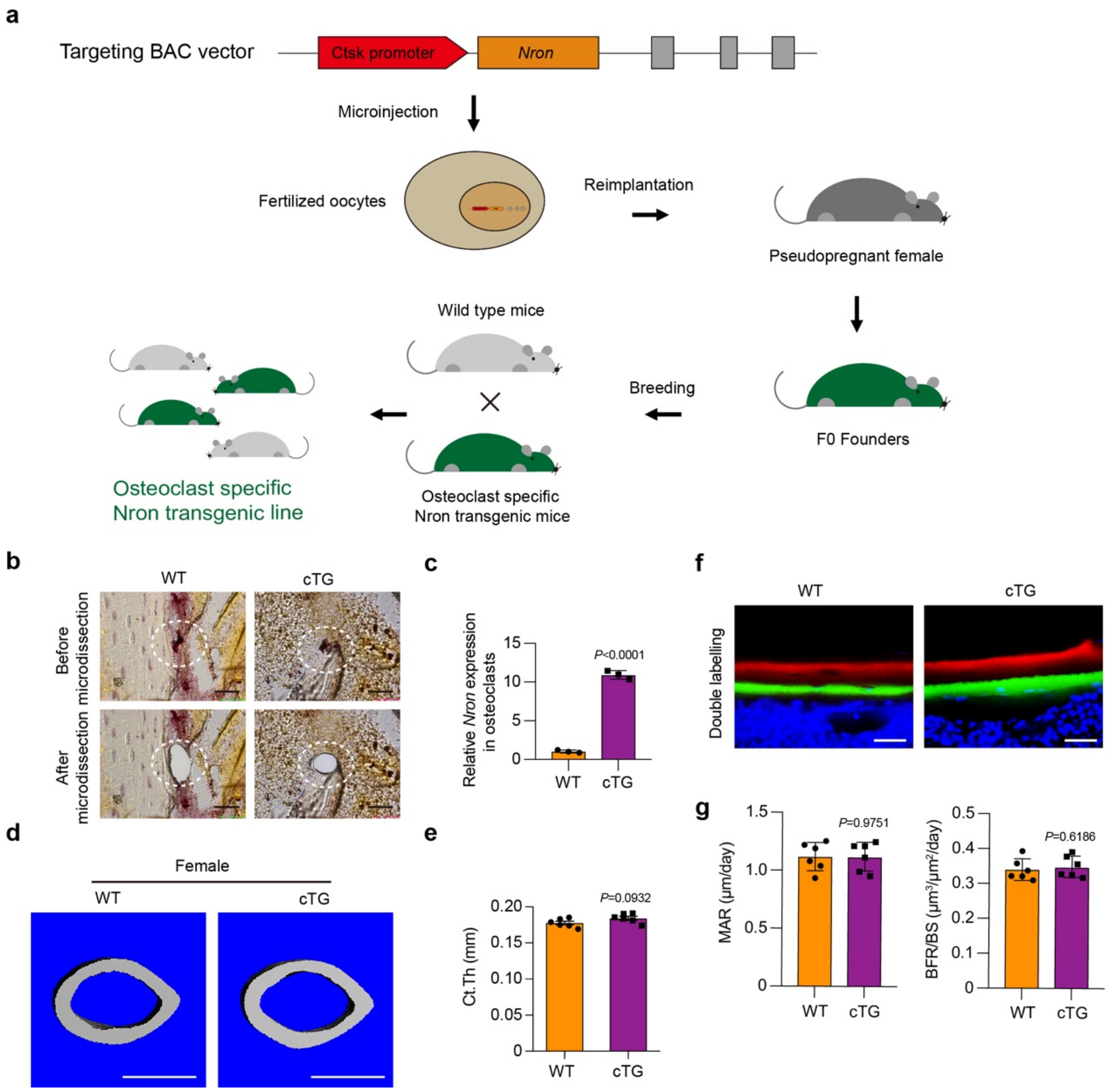

Supplementary Fig. 3 Generation and characterization of osteoclast-specific Nron transgenic mice. (**a**) Schematic illustration of the generation of osteoclast-specific Nron transgenic mice. (**b**) Representative images of the TRAP+ osteoclasts in femur from 6-month-old female WT and Nron cTG mice before and after the laser-capture microdissection, (similar results were obtained in all mice, n=3/group). Scale bar: 25 μm. (**c**) Q-PCR analysis of *Nron* expression in TRAP+ osteoclasts isolated by laser-capture microdissection from 6-month-old female WT and Nron cTG mice, (n=3/group). **(d)** Representative μCT images showing the 3D cortical bone structures of the femurs from 6-month-old female WT and Nron cTG mice, (similar results were obtained in all mice, n=6/group). Scale bar: 1 mm. (**e**) μCT measurements of Ct.Th in femurs from 6-month-old female WT and Nron cTG mice, (n=6/group). **(f)** Representative images of bone formation rates of the 6-month-old female WT and Nron cTG mice as determined by double labeling of xylenol orange and calcein, (similar results were obtained in all mice, n=6/group). Scale bar: 20 μm. **(g)** Bone histomorphometric analysis of MAR and BFR/BS in femurs from 6-month-old female WT and Nron cTG, (n=6/group). Data are presented as the means ± s.e.m. Significant differences between two groups were determined by unpaired two-tailed Student's t-test. Source data are provided as a Source Data file.

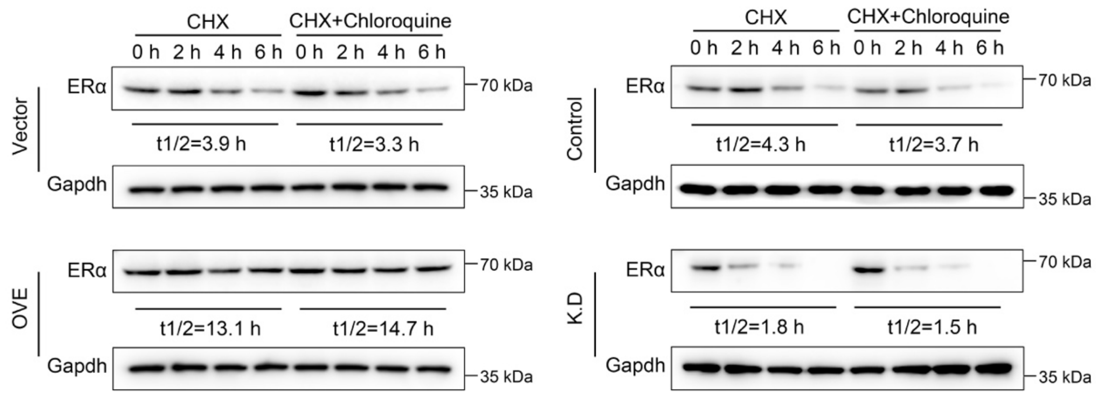

Supplementary Fig. 4 The protein stability (t1/2) and lysosomal-mediated degradation of ERα assessed by measuring the decline of protein levels after CHX (10 μg/ml) or CHX (10 μg/ml) + chloroquine (50 μM) treatment in osteoclasts. The experiment was repeated three times independently with similar results. Source data are provided as a Source Data file.

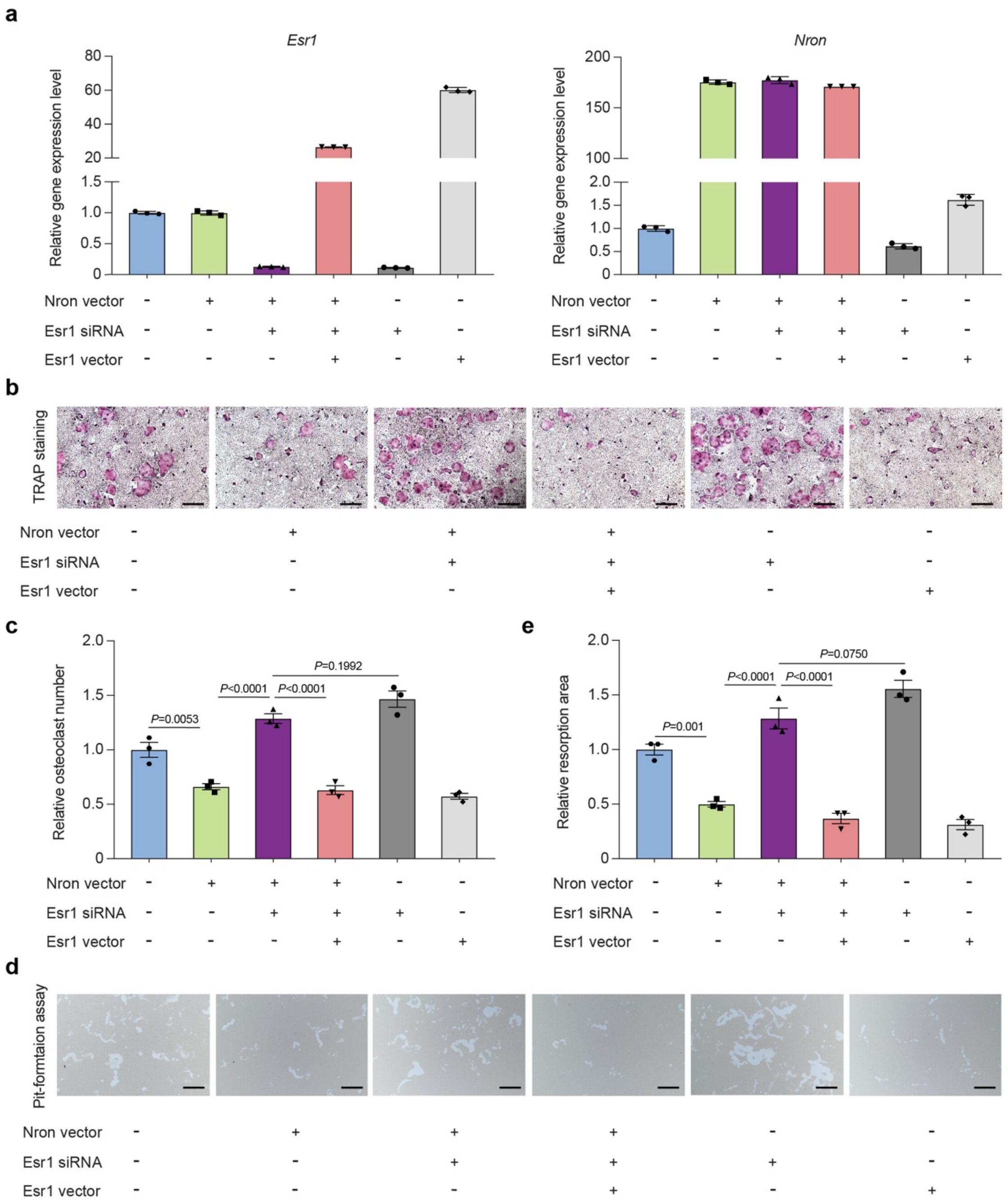

Supplementary Fig. 5 ERα was a mediator of the anti-osteoclastic effects for Nron. The osteoclasts were transfected with Esr1 siRNAs or Esr1 and Nron overexpression vectors with the combinations indicated in the figure to mediated the gene overexpression or knockdown at day 4 after mouse BMMs osteoclast differentiation. Then, the osteoclasts were further cultured for two days in the M-CSF+RANKL osteoclast differentiation induction medium in the presence of 10 nM estradiol. Then, the cells were collected for subsequent Q-PCR, TRAP staining and Pit-formation assays. (**a**) Q-PCR analysis of the gene knockdown or overexpression efficiency of Esr1 and Nron, (n=3 biologically independent experiments). (**b**) Representative TRAP staining images of the osteoclasts generated in different groups. The experiment was repeated three times independently with similar results. Scale bar: 500 μm. (**c**) Quantification of the relative osteoclast number per well in different groups, (n=3 biologically independent experiments). (**d**) Representative images of the osteoclast resorption pits in Corning Osteo Assay Surface Plate in different groups. The experiment was repeated three times independently with similar results. Scale bar: 200 μm. (**e**) Quantification of the relative osteoclast resorption area per well in different groups, (n=3 biologically independent experiments). Data are presented as the means ± s.e.m. Significant differences among multiple groups were determined by one-way ANOVA with Tukey's multiple comparisons test. Source data are provided as a Source Data file.

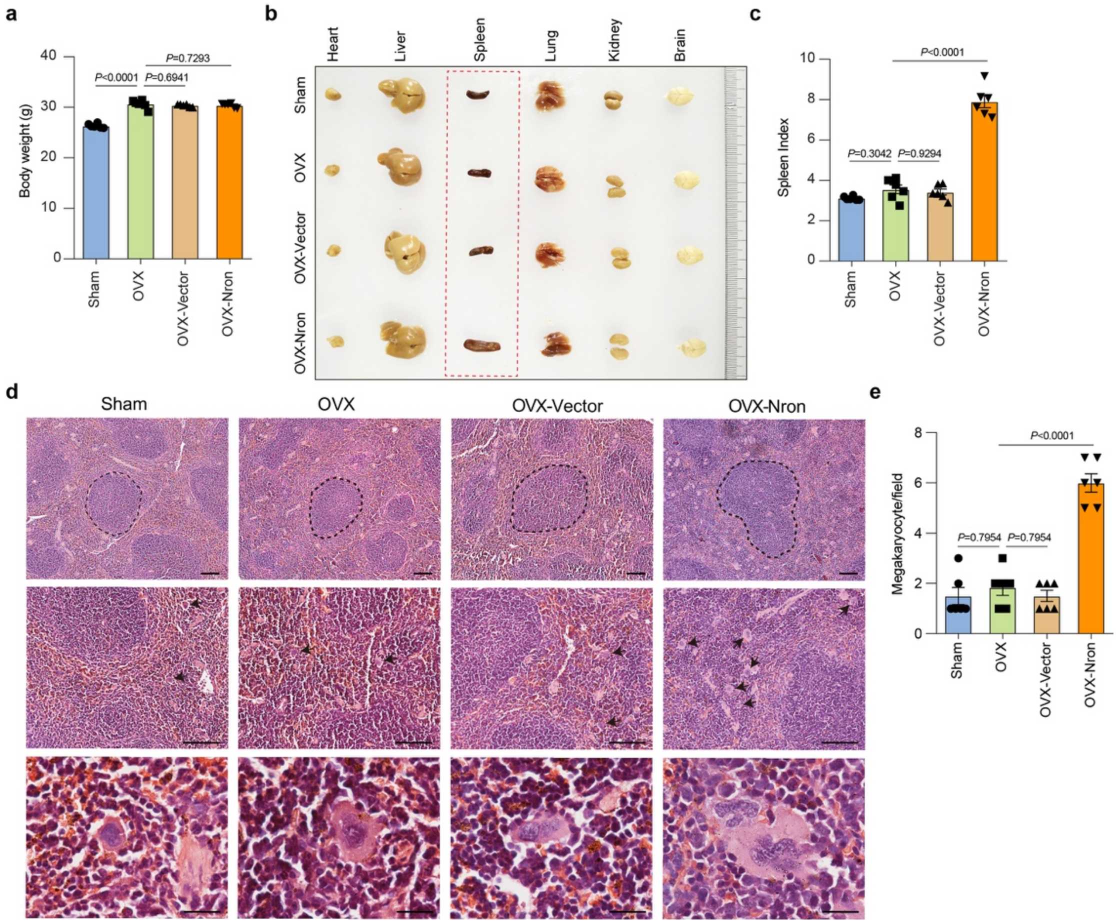

Supplementary Fig. 6 Osteoclastic delivery of Nron caused splenomegaly. (**a**) Body weight of the mice from Sham, OVX, OVX-Vector and OVX-Nron group, (n=6/group). (**b**) General images of main organs of mice from Sham, OVX, OVX-Vector and OVX-Nron group. (**c**) Spleen index (spleen weight (mg) divided by body weight (g)) of mice from Sham, OVX, OVX-Vector and OVX-Nron group, (n=6/group). (**d**) Representative H&E staining images of spleen from Sham, OVX, OVX-Vector and OVX-Nron group, (similar results were obtained in all mice, n=6/group). Scale bar: 500 μm (upper); 100 μm (middle); 20 μm (lower). Black arrow indicates megakaryocytes. (**e**) Quantification of megakaryocyte in (d), (n=6/group). Data are presented as the means ± s.e.m. Significant differences among multiple groups were determined by one-way ANOVA with Dunnett's multiple comparisons test. Source data are provided as a Source Data file.

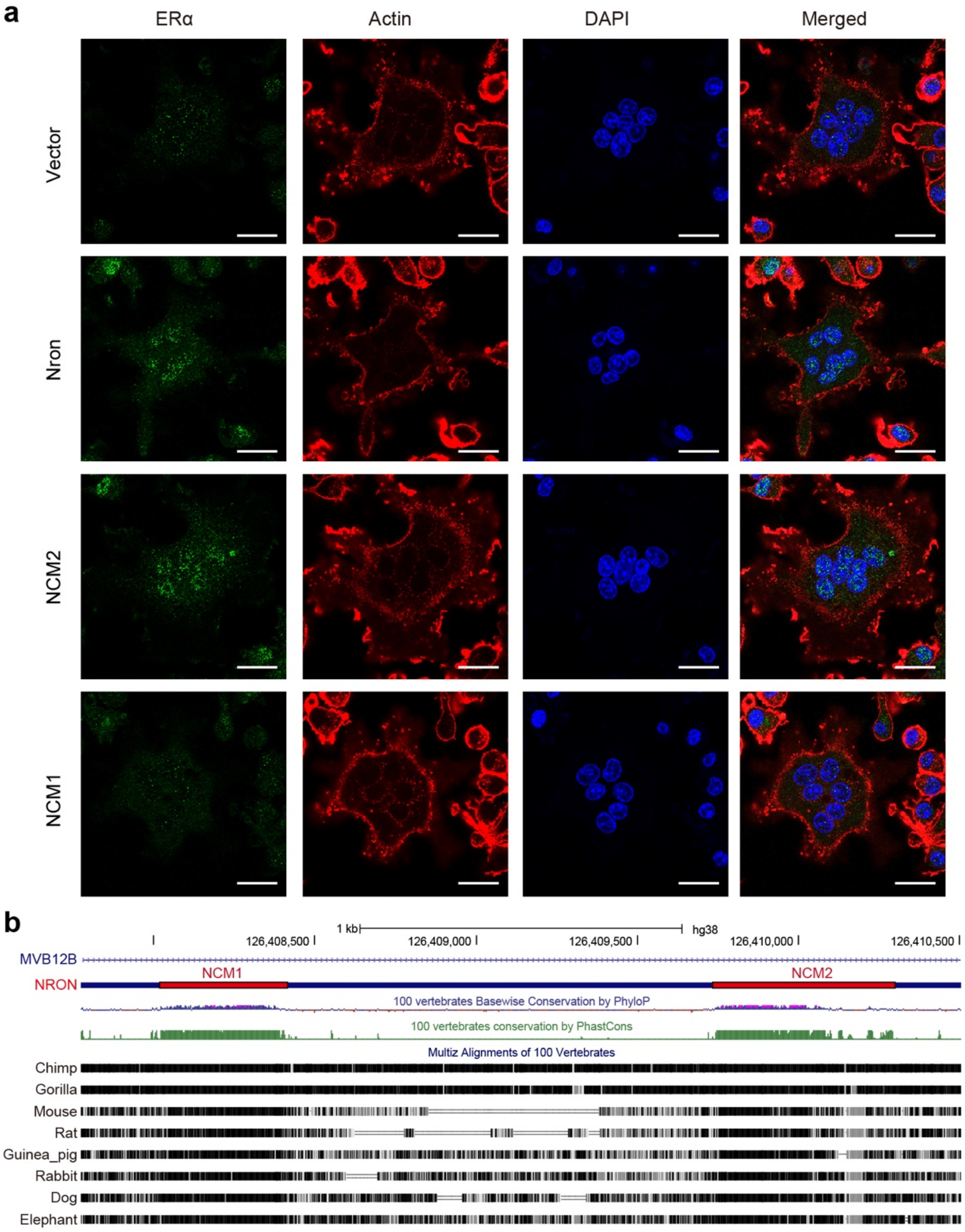

Supplementary Fig. 7 Nron modulate ERα expression in osteoclast. (**a**) Representative confocal images of ERα in osteoclast with Nron, NCM1, NCM2 overexpression. Scale bar: 20 μm. The experiment was repeated three times independently with similar results. (**b**) Genome browser depiction of NRON and its conserved analogs in multiple species.

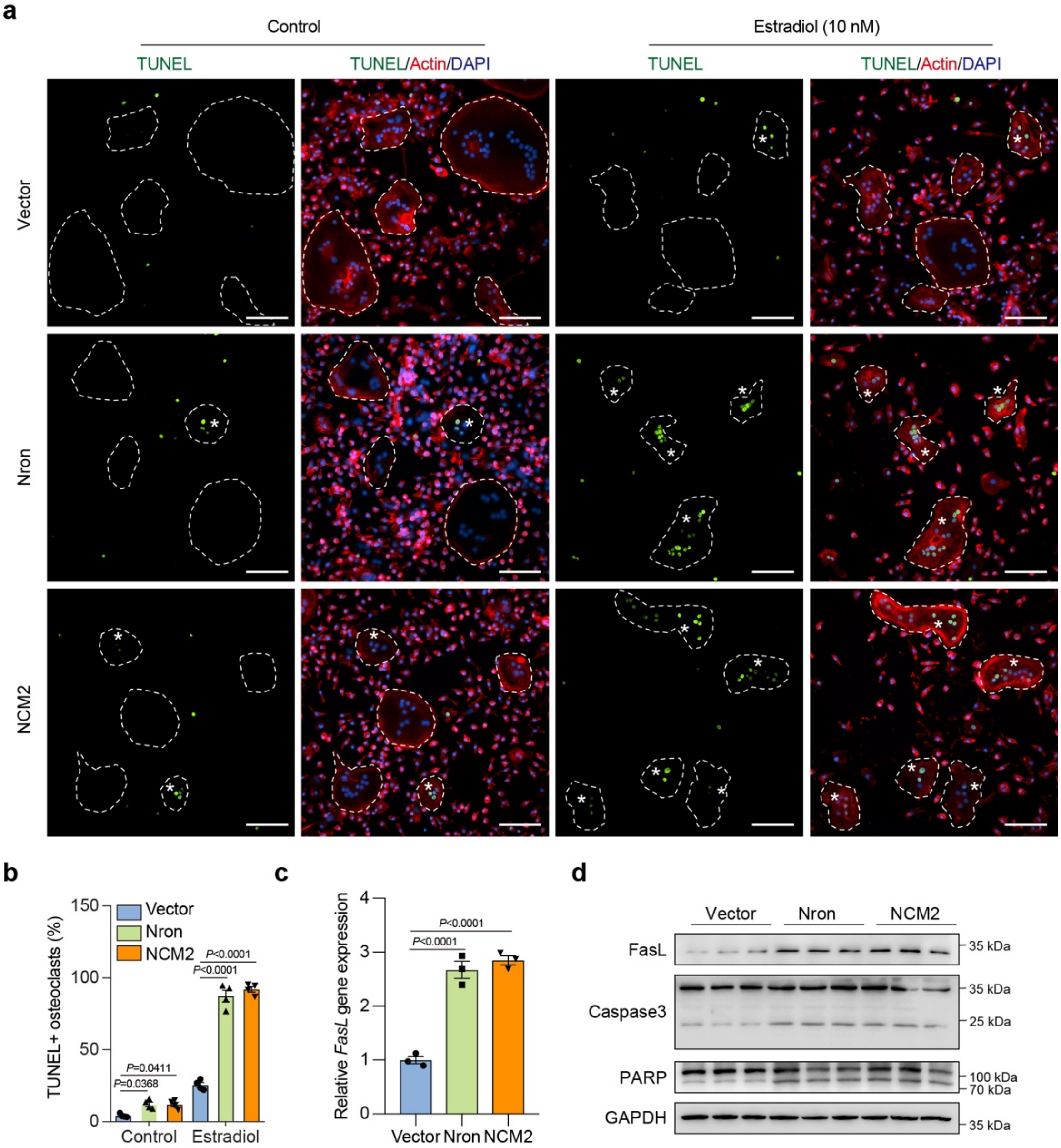

Supplementary Fig. 8 Nron and NCM2 modulate osteoclast survival. (**a**) Representative TUNEL staining images of osteoclast with or without estradiol (10 nM) treatment for 48 h in vector, Nron or NCM2 overexpression groups. The asterisk indicates the TUNEL+ apoptotic osteoclast cells. The experiment was repeated four times independently with similar results. Scale bar: 100 μm. (**b**) Quantification statistics of the TUNEL+ apoptotic osteoclast cells in (a), (n=4 biologically independent experiments). (**c**) Expressions of *FasL* gene in osteoclasts with Nron or NCM2 overexpression treatment with estradiol (10 nM) treatment for 48 h analyzed by Q-PCR. (n=3 biologically independent experiments). (**d**) Expressions of FasL and apoptosis markers Caspase 3 and PARP in osteoclasts with Nron or NCM2 overexpression treatment with estradiol (10 nM) treatment for 48 h analyzed by Western-blot (WB). The experiment was repeated three times independently with similar results. Data are presented as the means ± s.e.m. The significant differences among multiple groups were determined by two-way ANOVA (b) with Sidak's multiple comparisons test or one-way ANOVA with Dunnett's multiple comparisons test (c). Source data are provided as a Source Data file.

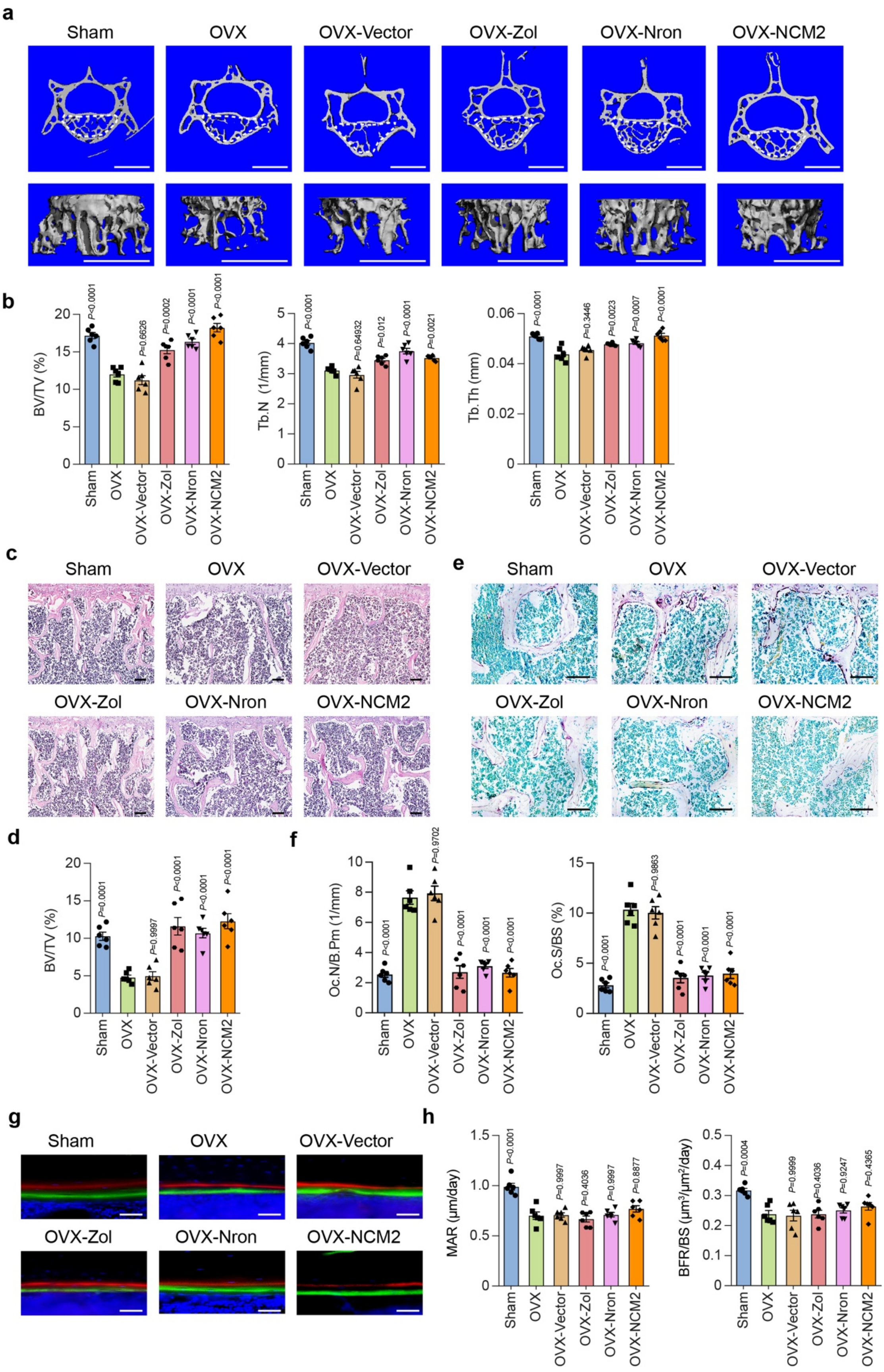

Supplementary Fig.9 See next page for caption

Supplementary Fig.9 Osteoclastic delivery of NCM2 increases vertebrae bone mass in osteoporotic mice. (**a**) Representative μCT images showing the 3D bone structures of mice fifth lumbar vertebrae from Sham, OVX, OVX-Vector, OVX-Zol, OVX-Nron and OVX-NCM2 group, (similar results were obtained in all mice, n=6/group). Scale bar: 1 mm (all panels). (**b**) μCT measurements of BV/TV, Tb.N and Tb.Th in fifth lumbar vertebrae from Sham, OVX, OVX-Vector, OVX-Zol, OVX-Nron and OVX-NCM2 group, (n=6/group). (**c**) Representative H&E staining images of mice fifth lumbar vertebrae from Sham, OVX, OVX-Vector, OVX-Zol, OVX-Nron and OVX-NCM2 group, (similar results were obtained in all mice, n=6/group). Scale bar: 100 μm. (**d**) Histological measurements of BV/TV values in (c), (n=6/group). (**e**) Representative TRAP staining images of mice fifth lumbar vertebrae from Sham, OVX, OVX-Vector, OVX-Zol, OVX-Nron and OVX-NCM2 group, (similar results were obtained in all mice, n=6/group). Scale bar: 100 μm. (**f**) Quantification of osteoclastic metrics Oc.N/B.Pm and Oc.S/BS in (e), (n=6/group). **(g)** Representative images of bone formation rates of femur from Sham, OVX, OVX-Vector, OVX-Zol, OVX-Nron and OVX-NCM2 group as determined by double labelling of xylenol orange and calcein, (similar results were obtained in all mice, n=6/group). Scale bar: 20 μm. **(h)** Bone histomorphometric analysis of MAR and BFR/BS in (g), (n=6/group). Data are presented as the means ± s.e.m. The significant differences between Sham, OVX-Vector, OVX-Zol, OVX-Nron, OVX-NCM2 group and OVX group were determined by one-way ANOVA with Dunnett's multiple comparisons test. Source data are provided as a Source Data file.

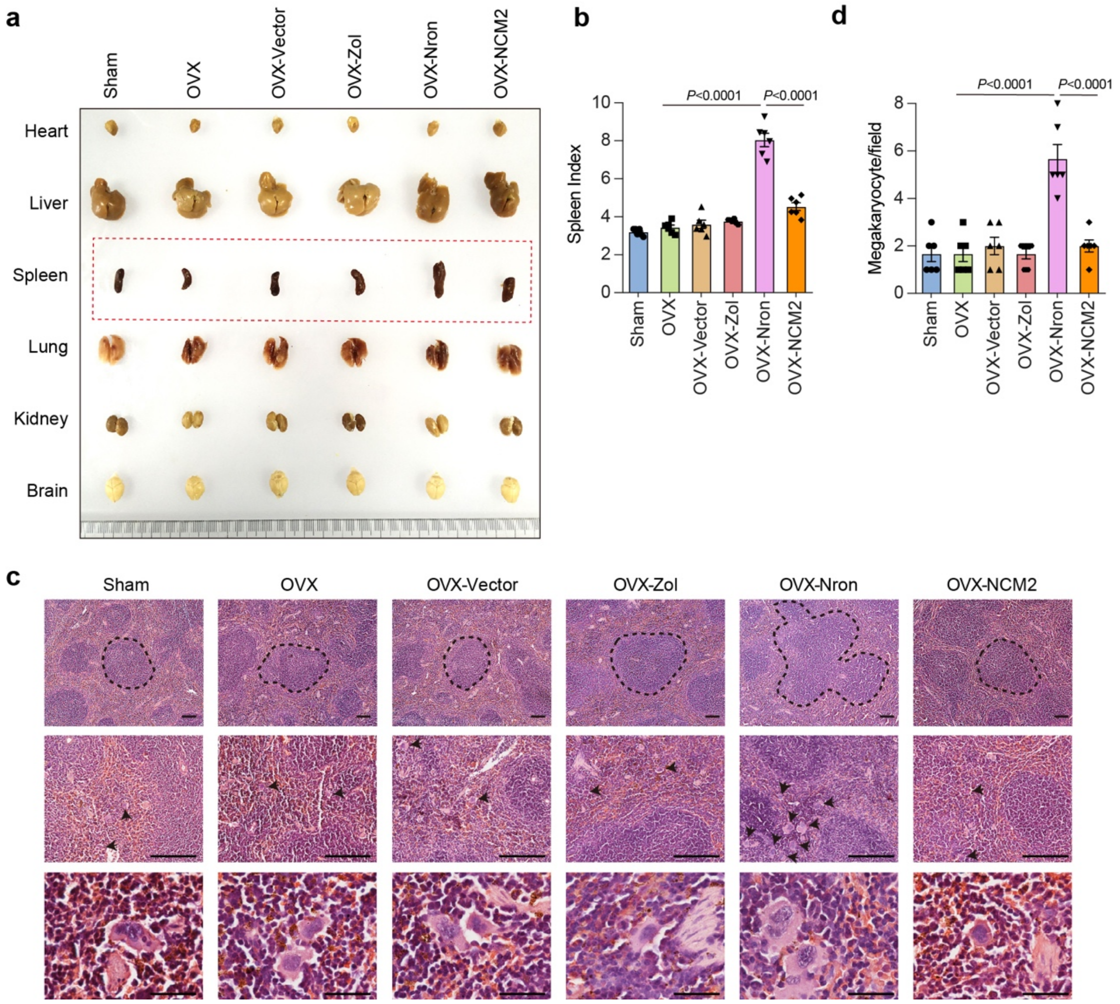

Supplementary Fig. 10 Safety evaluation of Nron functional motif therapy. (**a**) General images of main organs of mice from Sham, OVX, OVX-Vector, OVX-Zol, OVX-Nron and OVX-NCM2 group. (**b**) Spleen index of mice from Sham, OVX, OVX-Vector, OVX-Zol, OVX-Nron and OVX-NCM2 group, (n=6/group). (**c**) Representative H&E staining images of spleen from Sham, OVX, OVX-Vector, OVX-Zol, OVX-Nron and OVX-NCM2 group, (similar results were obtained in all mice, n=6/group). Scale bar: 500 μm (upper); 100 μm (middle); 20 μm (lower). Black arrow indicates megakaryocytes. (**d**) Quantification analysis of megakaryocyte in (c), (n=6/group). Data are presented as the means ± s.e.m. The significant differences between multiple groups were determined by one-way ANOVA with Tukey's multiple comparisons test. Source data are provided as a Source Data file.

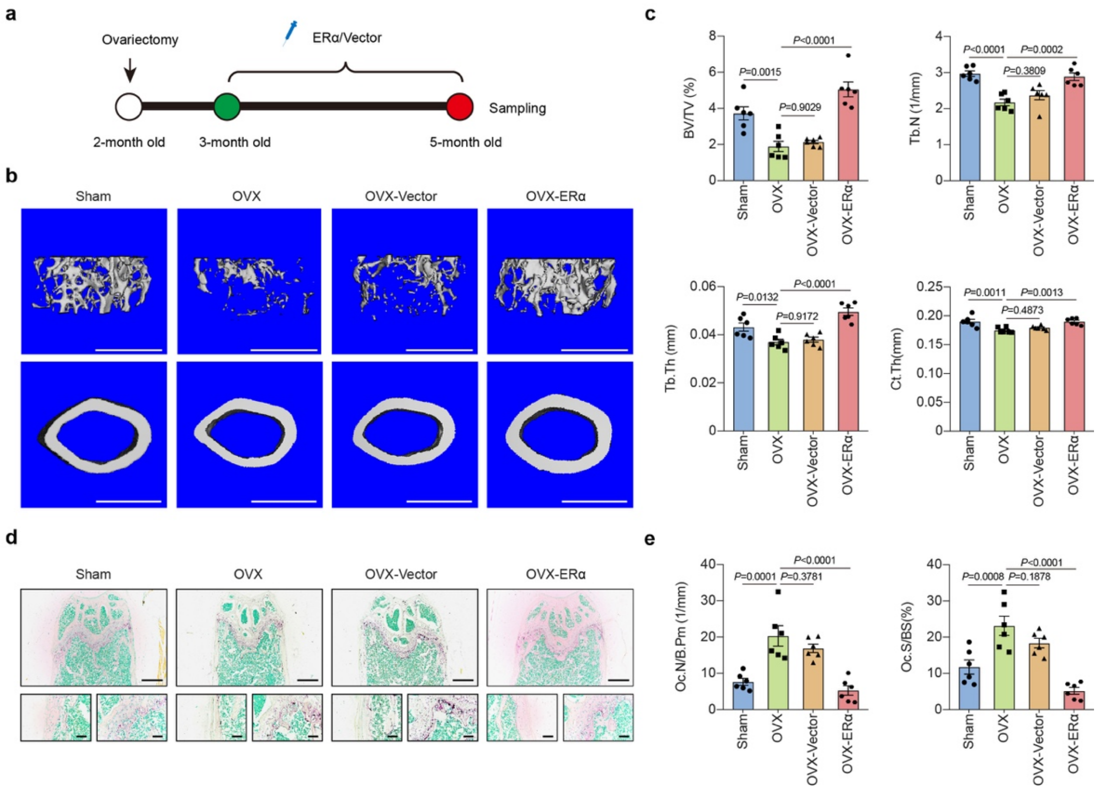

Supplementary Fig. 11 Osteoclastic delivery of ERα increases bone mass in OVX mice. (**a**) Schematic diagram illustrating the experimental design of ERα treatment in OVX mice. (**b**) Representative µCT images showing the 3D bone structures of mice femurs from Sham, OVX, OVX-Vector and OVX-ERα group, (similar results were obtained in all mice, n=6/group). Scale bar: 1 mm (all panels). (**c**) µCT measurements of BV/TV, Tb.N, Tb.Th and Ct.Th in femurs from Sham, OVX, OVX-Vector and OVX-ERα group, (n=6/group). (**d**) Representative TRAP staining images of mice femurs from Sham, OVX, OVX-Vector and OVX-ERα group, (similar results were obtained in all mice, n=6/group). Scale bar: 500 µm (upper panels); 100 µm (lower panels). (**e**) Quantification of osteoclastic metrics Oc.N/B.Pm and Oc.S/BS in (d), (n=6/group). Data are presented as the means ± s.e.m. The significant differences between Sham, OVX-Vector, OVX-ERα treatment group with OVX group were determined by one-way ANOVA with Dunnett's multiple comparisons test. Source data are provided as a Source Data file.

Supplementary Table 1. Primers used for Q-PCR.

**Primer name**	**Primer sequence**
Human *GAPDH* F	AGCCTCAAGATCATCAGCAATG
Human *GAPDH* R	CACGATACCAAAGTTGTCATGGAT
Human *NRON* F	TGCATGCTGGCTTTCTGTCT
Human *NRON* R	CATTCTAGTCCCAGCTCCGC
Mouse *Gapdh* F	AGGTCGGTGTGAACGGATTTG
Mouse *Gapdh* R	TGTAGACCATGTAGTTGAGGTCA
Mouse *Nron* F	TTCAAACGGCTTAGCACCCT
Mouse *Nron* R	TGTCCTGCTGGGACCAGATA
Mouse *Esr1* F	ACCATTGACAAGAACCGGAG
Mouse *Esr1* R	CCTGAAGCACCCATTTCATT
Mouse *Cul4b* F	GAGCCGCTTTTACCAGCAAG
Mouse *Cul4b* R	AGCTTTACCATCTCTGGCGG
Mouse *FasL* F	GGGTCTACTTACTACCTCAC
Mouse *FasL* R	CCCTCTTACTTCTCCGTTA
Mouse *H19* F	ATGCCCTAACCGCTCAGTCC
Mouse *H19* R	CTCACCACCCCAGCTACTCG
Mouse *D130058E05Rik* F	CTATTCAGGGGAAGGCGGGG
Mouse *D130058E05Rik* R	CGGGTAGGTAGCAAGGGTCC
Mouse *Gm45441* F	AAGACCTTGCCTGAGCCGATG
Mouse *Gm45441* R	TGAAAGGTGTTCCTGGAGGGC
Mouse *Rapgef4os* F	ACCTGGGGTGGATGCTCAAG
Mouse *Rapgef4os* R	GTAGTTGGGCTCTCCCCATCC
Mouse *Ino80dos* F	AAAAAGTGCTCCCAGGGTCGG
Mouse *Ino80dos* R	GTTATGTCCTCGCGGTGCCT
Mouse *Zeb2os* F	GGAGAACTCCAAGGGGCGAA
Mouse *Zeb2os* R	ACGTGCACCTCAAACCTAGTCA
Mouse *AI480526* F	ACAGGTCTTGGCCGGAGATAC
Mouse *AI480526* R	CAGTCAAATTGGGCCTGCTCC

Sequences

Sequence of human NRON functional motif NCM2

AACTTTGAAACTTTCCTTTGTATATTACATTAATCCATATAAGAAAATCTCTTTTAATGAGGAGTGATGTAGTTACAAA

CTCTCCAGAGCCATAATTAGAGCAAACTAATTGAAGTTGTGTTTTGACACACTTTCCAAATTCACGGGTGCCGGAT

GACATATTCACAACACTGCTGCCGCACAACCATGGCGACGGCAAAATCATTAGGCTAATAACGCTTATTTGCATTC

TTATCATGCCGGCAGCTCGCCCTTAAATACTGTTTCCACTACTGCTCCTTTACTGTAAGTTTCCACCGAAAGATATT

AACAGTAATTATTAGTACTTTAGTGGAATTTTAATGTTAA

Sequence of mouse Nron functional motif NCM2

AACTTTGAAACTTTCCTTTGTATATTACATTAATCCACATAAGAAAATCTCTTTTAATGAGGAGTGATGTAGTCACAAA

CTCTCCAGAGCCATAATTAGAGCAAACTAATTGAAGTTGTGTTTTGACACACTTTCCAAATTCACGGGTGCTGGAT

GACATATTCACAACACTGGTGCCGCACAACCATGGCGACGGCAGAATCATTAGGCTAATAACGCTTATTTGCATTC

TTCTCATGCCGCCAGCTCTCCCTTAAATACTGTTTCTACCACTGCTCCTTTACTGTAACTTTCCACTCGACGGTATT

AACAGTAATTATTAGTGCTTTAGTGGAATTTTAATGTTAAGAATCA
