# Supplementary material for: A functional motif of long noncoding RNA Nron against osteoporosis
Source: Nat Commun. 2021 Jun 3;12:3319. doi: 10.1038/s41467-021-23642-7 (PMC8175706; doi:10.1038/s41467-021-23642-7)
Supplement: Supplementary file 2 — Reporting Summary [file 41467_2021_23642_MOESM2_ESM.pdf]

nature research

Corresponding author(s): Yifei Wang, Yao Sun, Xiaogang Wang

Last updated by author(s): Mar 22, 2021

Reporting Summary

Nature Research wishes to improve the reproducibility of the work that we publish. This form provides structure for consistency and transparency in reporting. For further information on Nature Research policies, seeAuthors & Referees and theEditorial Policy Checklist .

Statistics

For all statistical analyses, confirm that the following items are present in the figure legend, table legend, main text, or Methods section.

n/a	Confirmed	
☐	☒	The exact sample size (*n*) for each experimental group/condition, given as a discrete number and unit of measurement
☐	☒	A statement on whether measurements were taken from distinct samples or whether the same sample was measured repeatedly
☐	☒	The statistical test(s) used AND whether they are one- or two-sided<br>*Only common tests should be described solely by name; describe more complex techniques in the Methods section.*
☒	☐	A description of all covariates tested
☒	☐	A description of any assumptions or corrections, such as tests of normality and adjustment for multiple comparisons
☐	☒	A full description of the statistical parameters including central tendency (e.g. means) or other basic estimates (e.g. regression coefficient) AND variation (e.g. standard deviation) or associated estimates of uncertainty (e.g. confidence intervals)
☐	☒	For null hypothesis testing, the test statistic (e.g. *F*, *t*, *r*) with confidence intervals, effect sizes, degrees of freedom and *P* value noted<br>*Give P values as exact values whenever suitable.*
☒	☐	For Bayesian analysis, information on the choice of priors and Markov chain Monte Carlo settings
☒	☐	For hierarchical and complex designs, identification of the appropriate level for tests and full reporting of outcomes
☒	☐	Estimates of effect sizes (e.g. Cohen's *d*, Pearson's *r*), indicating how they were calculated

Our web collection on statistics for biologists contains articles on many of the points above.

Software and code

Policy information about availability of computer code

Data collection	RNA sequencing was performed in Illumina HiSeqTM 2500 (Illumina, USA). The micro-CT data were collected using a micro-CT system (µCT50, Scanco Medical, Switzerland).
Data analysis	Bowtie2 (v.2.2.6) was used for removing rRNA mapped reads. The remaining reads were then mapped to the reference genome of mm10 using Tophat2 (v. 2.1.1). The reconstruction of transcripts was carried out with Cufflinks software (v.1.2.1) and transcripts abundances were quantified by using RSEM (v1.3.1) software. A differential expression analysis was performed using EdgeR (v3.12.0). The conservation of 200-bp sequences near each site of DElncRNAs was calculated respectively using the programs of PhastCons and PhyloP in software of PHAST (v.1.6.9), based on the known phylogenetic tree structure and phylo-HMM. The phylogenetic tree was constructed with neighbor-joining method using MEGA7 (v.7.0.21) software. The highly conserved motif was discovered with MEME (v.4.9.1) software (http://meme-suite.org/tools/meme). The micro-CT image data were reconstructed and analyzed using Mimics software (v.13.0, Materialise NV). The bone statistical histomorphometric analyses were performed using Bioquant Osteo software (v.7.20.10, Bioquant Nashville). The osteoclast resorption area per well was analyzed by image analysis freeware ImageJ (v.1.52a, National Institutes of Health, USA). The protein density was quantified using Quantity One Software (v4.62, Bio-Rad). All statistical analyses were performed with GraphPad Prism (v.8.0).

For manuscripts utilizing custom algorithms or software that are central to the research but not yet described in published literature, software must be made available to editors/reviewers. We strongly encourage code deposition in a community repository (e.g. GitHub). See the Nature Research guidelines for submitting code & software for further information.

Data

Policy information about availability of data

All manuscripts must include a data availability statement. This statement should provide the following information, where applicable:

- Accession codes, unique identifiers, or web links for publicly available datasets
- A list of figures that have associated raw data
- A description of any restrictions on data availability

The authors declare that the data supporting the findings of this study are available within the paper and its Supplementary information files. Any remaining data that support the results of the study will be available from the corresponding author upon reasonable request. Source data for Figs. 1-8 and Supplementary Figs. 1-6, Figs. 8-11 have been provided in the Source Data File. The high-throughput RNA-seq data have been have been deposited in the Gene Expression Omnibus (GEO) under accession code GSE134457 [https://www.ncbi.nlm.nih.gov/geo/query/acc.cgi?acc=GSE134457].The datasets generated during and/or analyzed during the current study are available from the corresponding author on reasonable request. The highly conserved sequences in different species (Mouse, Rat, Human, Chimp, Gorilla, Rabbit, Horse, Guinea Pig, Dog, Elephant) was obtained from UCSC (http://genome.ucsc.edu) and Ensemble (http://asia.ensembl.org/Multi/Tools/Blast).

Field-specific reporting

Please select the one below that is the best fit for your research. If you are not sure, read the appropriate sections before making your selection.

☒ Life sciences ☐ Behavioural & social sciences ☐ Ecological, evolutionary & environmental sciences

For a reference copy of the document with all sections, see nature.com/documents/nr-reporting-summary-flat.pdf

Life sciences study design

All studies must disclose on these points even when the disclosure is negative.

Sample size	No statistical analysis was used to predetermine sample sizes. Sample sizes were decided based on the relevant publications cited in the manuscript such as (Nat. Med. 24, 667-678, (2018), Cell Death Differ. 26, 2358-2370, (2019), J. Clin. Invest. 128, 5251-5266, (2018)) and prior experience in our laboratory (Nat. Metab. 1, 485-496, (2019), Nat. Med. 1, 93-100, (2013)). For in vitro analysis, at least 3 biological replicates were used for experiments. For WB, RNA-pull down assay and RNA immunoprecipitation assay, the experiment was repeated three times independently with similar results. For animal experiments, 6 mice were included in each group.
Data exclusions	For in vitro tests, data that were drastic outliers due to technical variability were excluded. For animal samples, no data was excluded.
Replication	No replicates were performed for RNA-sequencing (when we finished RNA-sequencing, we performed Q-PCR assay to validate the target genes screening out by RNA-sequencing and found very good reproducibility, so we did not repeat the RNA-sequencing experiment again). Other experiments in this study were independently repeated at least three times, the numbers of replicates for each experiment were presented in the Figure legend. We confirm that all attempts at replication were successful.
Randomization	For animal samples, all samples were randomly numbered and deallocated into groups. For in vitro experiments, allocation of the different experimental treatments (e.g. gene transfection, drug treatment) occurred randomly. In brief, cells were plated and plates were randomly assigned to experimental or control groups.
Blinding	Investigators in this study are blinded to groups of mice samples from micro-CT and histological analysis. For in vitro experiments (eq. gene transfection, drug treatment, gel loading for western blots) the experimenters were not blinded to allocation because these experiments required that the investigators were not blinded. However, the person who perform measurements was not informed about the goals of the study and the nature of the treatment groups.

Reporting for specific materials, systems and methods

We require information from authors about some types of materials, experimental systems and methods used in many studies. Here, indicate whether each material, system or method listed is relevant to your study. If you are not sure if a list item applies to your research, read the appropriate section before selecting a response.

Materials & experimental systems

n/a	Involved in the study	
☐	☒	Antibodies
☐	☒	Eukaryotic cell lines
☒	☐	Palaeontology
☐	☒	Animals and other organisms
☒	☐	Human research participants
☒	☐	Clinical data

Methods

n/a	Involved in the study	
☒	☐	ChIP-seq
☒	☐	Flow cytometry
☒	☐	MRI-based neuroimaging

Antibodies

Antibodies used	The primary antibodies used in WB assay included: anti-ERα (Invitrogen, #MA1-12692, 1:200), anti-CUL4B (Proteintech, #12916-1-AP, 1:1000), anti-Ubiquitin (CST, #43124, 1:1000), anti-FasL (abcam, #ab15285, 1:1000), anti-PARP (CST, #9532, 1:1000), anti-GAPDH (CST, #5174, 1:1000), anti-Caspase3 (CST, #9662, 1:1000), Anti-mouse IgG, HRP-linked Antibody (CST, #7076, 1:5000) and Anti-rabbit IgG, HRP-linked Antibody (CST, #7074, 1:6000).<br>For RIP assay anti-CUL4B (Proteintech, #12916-1-AP, 5μg), negative control IgG (5 μg, Millipore, #PP64B).<br>For IP assay: normal mouse IgG (Santa, #sc-2025, 2 μg), ERα primary antibody (Invitrogen, #MA5-13065, 2 μg).<br>For IF assay: Goat anti-Mouse Alexa Fluor 488 secondary antibody (Invitrogen, #A-11001, 1:1000), anti-ERα (Invitrogen, # MA5-13065, 1:100), anti-CTSK (Abcam, #ab19027,1:400), anti-TRAP (Abcam, #ab191406, 1:100).
Validation	All antibodies were obtained from commercial sources with reported validation by the manufacture or published papers.<br>anti-ERα (Invitrogen, #MA1-12692), [https://www.thermofisher.com/cn/zh/antibody/product/Estrogen-Receptor-alpha-Antibody-clone-TE111-5D11-Monoclonal/MA1-12692]<br>Goat anti-Mouse Alexa Fluor 488 secondary antibody (Invitrogen, #A-11001), [https://www.thermofisher.com/cn/zh/antibody/product/Goat-anti-Mouse-IgG-H-L-Cross-Adsorbed-Secondary-Antibody-Polyclonal/A-11001]<br>anti-CUL4B (Proteintech, #12916-1-AP), [https://www.ptgcn.com/products/CUL4B-Antibody-12916-1-AP.htm]<br>anti-Ubiquitin (CST, #43124), [https://www.cellsignal.com/products/primary-antibodies/ubiquitin-antibody/3933]<br>anti-PARP (CST, #9532), [https://www.cellsignal.com/products/primary-antibodies/parp-46d11-rabbit-mab/9532]<br>anti-GAPDH (CST, #5174), [https://www.cellsignal.com/products/primary-antibodies/gapdh-d16h11-xp-rabbit-mab/5174]<br>anti-Caspase3 (CST, #9662), [https://www.cellsignal.com/products/primary-antibodies/caspase-3-antibody/9662]<br>Anti-mouse IgG, HRP-linked Antibody (CST, #7076), [https://www.cellsignal.com/products/secondary-antibodies/anti-mouse-igg-hrp-linked-antibody/7076]<br>Anti-rabbit IgG, HRP-linked Antibody (CST, #7074), [https://www.cellsignal.com/products/secondary-antibodies/anti-rabbit-igg-hrp-linked-antibody/7074]<br>anti-FasL (abcam, #ab15285), [https://www.ncbi.nlm.nih.gov/pmc/articles/PMC7244041/, https://www.nature.com/articles/srep28347, https://www.jimmunol.org/content/191/11/5702.short]<br>anti-CTSK (Abcam, #ab19027), [https://www.nature.com/articles/s41467-017-02368-5, https://www.nature.com/articles/srep25198, https://rupress.org/jem/article-abstract/211/11/2249/41485]<br>negative control IgG (Millipore, #PP64B), [https://www.sigmaaldrich.com/catalog/product/mm/pp64?lang=zh&region=CN]<br>anti-TRAP (Abcam, #ab191406, 1:100), [https://www.abcam.com/tartrate-resistant-acid-phosphatase-antibody-epr15556-ab191406.html]<br>normal mouse IgG (Santa, #sc-2025), [https://www.scbt.com/p/normal-mouse-igg?requestFrom=search]

Eukaryotic cell lines

Policy information about cell lines

Cell line source(s)	Human THP-1 cells was obtained from the Chinese Academy of Sciences Cell Bank.
Authentication	THP-1 cells were authenticated using standard methods including morphology check by microscope and growth curve analysis.
Mycoplasma contamination	The cell line was tested negative for mycoplasma using the Sigma LookOut Mycoplasma PCR Detection Kit (#MP0035-1KT).
Commonly misidentified lines<br>(See ICLAC register)	No commonly misidentified cell lines were used.

Animals and other organisms

Policy information about studies involving animals; ARRIVE guidelines recommended for reporting animal research

Laboratory animals	The osteoclast specific Nron transgenic mice (Nron-cTG) on a C57BL/6J genetic background were generated by Cyagen Biosciences (Guangzhou, China). The femurs of 3-month old or 6-month old female Nron-cTG and WT littermate mice were used for bone mass measurement and histological experiments.<br>The Nron floxed mice on a C57BL/6J genetic background were generated by Shanghai Research Center for Model Organisms (Shanghai, China). Then F0 Nron floxed chimeric mice mice were crossed with Rosa26-FlpE knock-in mice (3-month-old, both sex, in C57BL/6J genetic background, obtained from (Cyagen Biosciences, Guangzhou, China)) to remove the PGK-Neo cassette to generate the floxed F1 mice. The Nron floxed F1 mice were crossed with Ctsk-Cre mice (3-month-old, both sex, in C57BL/6J genetic background, generated by Cyagen Biosciences, Guangzhou, China)) to obtain the osteoclast specific Nron knockout mice (Nron-cKO). The femurs of 3-month old and 6-month old female Nron-cKO and WT littermate mice were used for bone mass measurement and histological experiments.<br>3-month-old female C57/BL6J mice were performed with ovariectomy surgery (OVX) or received sham operation.
Wild animals	This study did not involve wild animals.
Field-collected samples	This study did not involve field-collected samples.

Ethics oversight	All procedures for mouse studies were approved by the Ethics Committee of Tongji University School of Stomatology.

Note that full information on the approval of the study protocol must also be provided in the manuscript.
